# Identification of key lncRNAs associated with oxaliplatin resistance in colorectal cancer cells and isolated exosomes: From *In-Silico* prediction to *In-Vitro* validation

**DOI:** 10.1371/journal.pone.0311680

**Published:** 2024-10-14

**Authors:** Roxana Sahebnasagh, Hoda Deli, Amir Shadboorestan, Zeynab Vakili-Ghartavol, Najmeh Salehi, Tahereh Komeili-Movahhed, Zahra Azizi, Mohammad Hossein Ghahremani

**Affiliations:** 1 Department of Molecular Medicine, School of Advanced Technologies in Medicine, Tehran University of Medical Sciences, Tehran, Iran; 2 Department of Toxicology, Faculty of Medical Sciences, Tarbiat Modares University, Tehran, Iran; 3 School of Biology, College of Science, University of Tehran, Tehran, Iran; 4 Cellular and Molecular Research Center, Qom University of Medical Sciences, Qom, Iran; 5 Department of Toxicology and Pharmacology, Faculty of Pharmacy, Tehran University of Medical Sciences, Tehran, Iran; Fudan University, CHINA

## Abstract

One of the critical challenges in managing colorectal cancer (CRC) is the development of oxaliplatin (OXP) resistance. Long non-coding RNAs (lncRNAs) have a crucial role in CRC progression and chemotherapy resistance, with exosomal lncRNAs emerging as potential biomarkers. This study aimed to predict key lncRNAs involved in OXP-resistance using *in-silico* methods and validate them using RT-qPCR methods in CRC cells and their isolated exosomes. Two public datasets, GSE42387 and GSE119481, were downloaded from the GEO database to identify differentially expressed genes (DEGs) and miRNAs (DEmiRNAs) associated with OXP-resistance in the HCT116 cell line. The analysis of GSE42387 revealed 210 DEGs, and GSE119481 identified 73 DEmiRNAs. A protein-protein interaction (PPI) network analysis of the DEGs identified 133 interconnected genes, from which the top ten genes with the highest degree scores were selected. By intersecting predicted miRNAs targeting these genes with the DEmiRNAs, 38 common miRNAs were found. Subsequently, 224 lncRNAs targeting these common miRNAs were predicted. LncRNA-miRNA-mRNA network were constructed and the top five lncRNAs with the highest degree scores were identified. Analysis using the Kaplan-Meier plotter database revealed that the key lncRNAs *NEAT1*, *OIP5-AS1*, and *MALAT1* are significantly associated with the overall survival of CRC patients. To validate these lncRNAs, OXP-resistant HCT116 sub-cell line (HCT116/OXR) was developed by exposing parental HCT116 cells to gradually increasing concentrations of OXP. Exosomes derived from both HCT116 and HCT116/OXR cells were isolated and characterized utilizing dynamic light scattering (DLS), transmission electron microscopy (TEM), and Western blotting. RT-qPCR confirmed elevated levels of *NEAT1*, *OIP5-AS1*, and *MALAT1* in HCT116/OXR cells and their exosomes compared to parental HCT116 cells and their exosomes. This study concludes that *NEAT1*, *OIP5-AS1*, and *MALAT1* are associated with the OXP-resistance in CRC. The high levels of these lncRNAs in exosomes of resistant cells suggest their involvement in intercellular communication and resistance propagation. This positioning makes them promising biomarkers for OXP-resistance in CRC.

## Introduction

Colorectal cancer (CRC) is the third most common diagnosed malignany and the second most cause of cancer-related death worldwide [1]. In 2020, approximately 1.9 million new cases of CRC were diagnosed and 935,000 deaths were occurred globally [2]. The incidence rate of CRC is increasing and is predicted to reach over 2.2 million new cases and 1.1 million deaths annually by 2030 [3]. The available treatment options for CRC include surgical intervention, radiotherapy, chemotherapy, immunotherapy, and targeted therapy [4, 5]. Cytotoxic chemotherapy is considered the cornerstone of CRC treatment [6].

Oxaliplatin (OXP), a third-generation platinum compound, is one of the main component in cytotoxic chemotherapy which commonly used in combination with other cytotoxic drugs as first-line therapy for CRC patients. It exhibits extensive anti-tumor activity in metastatic cancer and various cell lines through the formation of covalent platinum-DNA adducts, which inhibit DNA replication and cell growth, ultimately leading to cell death [5, 7, 8]. The DNA damage induced by OXP results in transient S phase delay and G2/M arrest in cell cycle progression and enhances cell apoptosis processes [9–11]. Despite its effectiveness, most cases of advanced CRC suffer from the development of OXP-resistance, which often results in treatment failure and poor overall survival (OS) [11, 12]. OXP-resistance occur through various mechanisms, including the regulation of cell death, detoxification, cellular influx/efflux, alterations in DNA adduct repair, and epigenetic modifications [13]. Discovering the molecular mechanisms of OXP-resistance and associated biomarkers will help develop new treatments designed to overcome such resistance and aid in identifying patients who are unlikely to benefit from treatment with OXP [11].

Recent studies have demonstrated that long non-coding RNAs (lncRNAs), which exceed 200 nucleotides in length, play crucial roles in regulating various cellular processes. LncRNAs can interact with RNA, DNA, and proteins to form complexes that modulate transcription, RNA stability, translation, and alternative splicing [14, 15]. It has been shown that the intricate interaction between lncRNAs and microRNAs (miRNAs) shows their significance in RNA regulation processes. Some lncRNAs act as competitive endogenous RNAs (ceRNAs), functioning as sponges for miRNAs within the tumor regulation network to regulate gene expression [16]. LncRNAs can function as oncogenes or tumor suppressors by modulating crucial signaling pathways through interactions with regulatory molecules [17–19]. These lncRNAs significantly contribute to the development of malignant behaviors in various cancers by regulating critical cellular processes such as cell proliferation, apoptosis, cell cycle, autophagy, metastasis, epithelial-mesenchymal transition (EMT), invasion, and chemotherapy resistance [20]. Recent studies have identified several lncRNAs that are notably contribute to CRC. For instance, *MALAT1*, *NEAT1*, *HOTAIR*, *Linc00152*, *CRNDE*, *GAS5*, *KCNQ1OT1*, *TUG1*, *MEG3*, *PVT1*, *MEIS1*, and *H19* have been shown to influence cancer initiation, progression, and resistance to drugs such as OXP and 5-fluorouracil (5-FU) [21–32]. LncRNAs also transcend cellular boundaries through encapsulation in extracellular vesicles, where they influence the functions of recipient cells. This encapsulation protects lncRNAs from degradation, thereby preserving their functional integrity during cell-to-cell transfer [33].

Exosomes, a type of extracellular vesicle typically ranging from 30 to 150 nm in diameter, originate from the endocytic pathway and are secreted into the extracellular environment by various cell types [34]. They selectively package biomolecular cargo and deliver it to adjacent or distant cells, thereby mediating cellular communication, modulating gene expression, and altering the phenotypes and biology of recipient cells [35, 36]. Interestingly, recent research indicates that exosomes secreted by drug-resistant cancer cells play a critical role in spreading resistance traits through their paracrine effects, enabling sensitive cancer cells to acquire drug resistance. Exosomes contribute to this mechanism by transferring various drug-resistance-associated materials, including proteins (such as P-glycoprotein, ABC transporters, and multidrug resistance-associated proteins), metabolites (such as TGF-β and prostaglandin E2), and nucleic acids (such as DNA, miRNAs, and lncRNAs) to drug-sensitive recipient cells [37, 38]. Through these transfers, exosomes protect cancer cells from the cytotoxic effects of chemotherapeutic agents by mediating alterations in signal transduction, inhibiting apoptosis, enhancing drug tolerance, forming tumor niches, regulating drug efflux pumps, promoting immune escape, enhancing DNA repair, and altering the tumor microenvironment (such as hypoxia, EMT, and angiogenesis) [37, 39, 40]. Recent studies have demonstrated that exosomes carrying specific lncRNAs can influence CRC progression and OXP resistance. For instance, Ren et al. reported that exosomal lncRNA *H19* contributes to OXP resistance in CRC by acting as a sponge for *miR-141* and activating the β-catenin pathway [41]. Similarly, Deng et al. demonstrated that exosomes secreted by cancer-associated fibroblasts (CAFs), enriched with the lncRNA CCAL, promote OXP resistance in CRC cells by upregulating β-catenin and inhibiting apoptosis [42]. Additionally, CAF-derived exosomal FOSL1 has been shown to enhance cell proliferation, stemness, and OXP resistance in CRC cells by activating ITGB4 [43].

Given the roles of lncRNAs and exosomes in cancer drug resistance, they hold promise as biomarkers for predicting CRC progression and responses to chemotherapy [41, 44–46]. This study hypothesizes that specific lncRNAs within exosomes contribute to OXP-resistance in CRC, aiming to identify and validate these lncRNAs through a combined *in-silico* and *in-vitro* approach. To achieve this, differentially expressed genes (DEGs) and miRNAs (DEmiRNAs) between parental and OXP-resistant CRC cells were identified from two datasets. Using *in-silico* methods, mRNA-miRNA-lncRNA networks were constructed and key lncRNAs based on specific criteria were selected. To validate these, an OXP-resistant CRC cell line was established and characterized and exosomes from the parental and resistant cells were isolated. The expression levels of the identified key lncRNAs were then evaluated using qRT-PCR in both the cells and their derived exosomes.

## Materials and methods

### Data acquisition

In this study, an initial search was performed in the Gene Expression Omnibus (GEO) database to obtain microarray and RNA-seq data related to differentially expressed genes (DEGs), miRNAs (DEmiRNAs), and lncRNAs (DElncRNAs) associated with acquired OXP-resistance in CRC. Ultimately, two publicly available datasets, GSE42387 and GSE119481, were selected based on their comprehensive data for parental (HCT116) cells and OXP-resistant HCT116 (HCT116/OXR) sub-cell. Although other datasets, such as GSE119603, also explore the gene expression profiles of OXP-resistant in HCT116 cells, we chose to focus on the GSE42387 and GSE119481 datasets. The reason for this selection is that these two datasets demonstrate a higher level of resistance in the HCT116/OXR cells to OXP, making them more suitable for our research objectives. The selection of these datasets was also based on several key criteria. Firstly, these datasets focus on the HCT116 cell line, a well-established model for studying drug resistance in CRC, allowing for direct comparison of results and enhancing the relevance of the findings to the current study. Secondly, the methods used to establish the HCT116/OXR sub-cell lines in these studies are consistent with those employed in the current study, involving a stepwise increase in OXP concentration. This consistency ensures that the resistance mechanisms observed are comparable and directly applicable. Additionally, the integration of data from microarray and RNA-seq technologies provides a comprehensive view by leveraging the strengths of each method while compensating for their respective limitations. This combined approach offers more robust and detailed results, which are valuable for both bioinformatics analyses and experimental design.

#### Bibliography of data sources

The GEO dataset GSE42387, generated by Jensen et al., contains mRNA expression profiles of HCT116 and HCT116/OXR sub-cell lines. The OXP-resistant cells were established by exposing the parental HCT116 cell line to stepwise increasing OXP concentrations over 241 days. To confirm drug resistance, an MTT assay was performed. The HCT116/OXR sub-cell lines were then recovered in a drug-free culture medium for two weeks. Subsequently, total RNA was extracted from both HCT116 and HCT116/OXR cells. Each set of samples was obtained in triplicate: control parental HCT116 cell lines (GSM1038651, GSM1038652, and GSM1038653) and HCT116/OXR sub-cell lines (GSM1038654, GSM1038655, and GSM1038656). This triplicate sampling ensured the robustness and reliability of the findings. Labeled cRNAs were then hybridized to the Agilent Whole Human Genome Microarray 4x44K G4112F, based on the GPL16297 platform, providing a comprehensive gene expression profile with 32,750 probes [47].

The GEO dataset GSE119481, generated by Gasiule et al., contains the miRNA expression profile of parental HCT116 cells and the HCT116/OXR sub-cell line. The OXP-resistant sub-cell line were generated by exposing the parental HCT116 cells to stepwise increasing OXP-concentrations over nine months until stable resistance cells were achieved. MTT assays confirmed the resistance of HCT116 to OXP, showing a 27.7-fold increase in HCT116/OXR sub-cell compared to the parental HCT116. Total RNA was extracted from both the HCT116 and HCT116/OXR cells. Each set of samples was obtained in triplicate: control parental HCT116 cell lines (GSM3375480, GSM3375481, and GSM3375482) and HCT116/OXR sub-cell lines (GSM3375477, GSM3375478, and GSM3375479). cDNA libraries were then prepared, and miRNAs were examined using high-throughput sequencing on an Illumina MiSeq instrument (platform GPL15520) [48].

#### Data preprocessing for the identification of DEGs and DElncRNAs from the GSE42387 dataset

The pre-processed and normalized gene expression matrix sourced from the microarray dataset GSE42387 was acquired. To analyze gene expression data and perform a pairwise comparison of DEGs and DElncRNAs between the parental HCT116 cells and HCT116/OXR sub-cell line, the R packages ‘limma’ version 3.52.2 within the R software version 4.2.1 were utilized. The criteria set to determine DEGs were │log2 fold change (FC) │> 1 and a p-value < 0.05. Visualizations were conducted using the ggplot2 version 3.3.6 and pheatmap version 1.0.12 R package. Principal component analysis (PCA) was performed using the Base R prcomp function, and gene annotations were conducted using data.table version1.14.4. Gene annotation and functional analysis were conducted using the Ensembl (https://asia.ensembl.org/index.html), GeneCard (https://www.genecards.org/) and NCBI (https://www.ncbi.nlm.nih.gov/) databases.

#### Data preprocessing for the identification of DEmiRNAs from the GSE119481 dataset

To obtain DEmiRNAs, the miRNA expression profiles from GSE119481 dataset were downloaded for subsequent analysis. Data analysis and visualization were conducted using R software v4.2.1 and multiple R packages, including DESeq2 (version 1.42.0), RColorBrewer (version 1.1.3), gplots (version 3.1.3), ggplot2 (version 3.4.0), and org.Hs.eg.db (version 3.18.0), to obtain a comprehensive list of DEmiRNAs. DESeq2, a user-friendly RNA-Seq data analysis package for handling high-dimensional count data, provides functionalities such as normalization, visualization and differential analysis [49]. The org.Hs.eg.db package was utilized for annotation, while visualizations were generated using the ggplot2, gplots, and RColorBrewer R package. The criteria set to determine DEmiRNAs.were │log2 fold change (FC)│> 0.6 and a p-value < 0.05.

### Construction of Protein-Protein Interaction (PPI) network

To explore the connected genes, a PPI network of DEGs was constructed using the Search Tool for the Retrieval of Interacting Genes (STRING) online databe version 12 (https://string-db.org) with a confidence score > 0.4 [50] and visualized using Cytoscape software (version 3.10.0) (https://cytoscape.org/) [51].

### Gene Ontology (GO) and pathway enrichment analysis

To explore the functions and pathways of the identified connected genes in the PPI network, the Database for Annotation, Visualization, and Integrated Discovery (DAVID) (https://david.ncifcrf.gov/home.jsp) was used to perform Gene Ontology (GO) annotation and Kyoto Encyclopedia of Genes and Genomes (KEGG) pathway analysis [52, 53]. A cutoff criterion of an EASE score < 0.05 was set, indicating significant enrichment.

### Screening central genes in PPI network

To identify the central nodes (genes) in the PPI network, the CytoNCA plug-in (version 2.1.6) in Cytoscape was used to calculate the degree scores of the gene nodes, representing the number of edges connecting the genes [54]. The top 10 genes with the highest degree scores were selected as the central genes for further analysis.

### Prediction of miRNAs targeting central genes

DIANA-TarBase version 9 (https://dianalab.e-ce.uth.gr/tarbasev9) and miRTarBase version 9 (https://mirtarbase.cuhk.edu.cn/) were used as data sources to predict upstream miRNAs targeting top10 genes based on experimentally validated miRNA-target interactions. These databases provide valuable insights into miRNA-mRNA interactions [55, 56]. Subsequently, an intersection was performed between these predicted miRNAs and the DEmiRNAs obtained from the GSE119481 dataset analysis. This process resulted in a list of overlapped miRNAs, from which miRNA-mRNA pairs were constructed.

### Prediction and enrichment analysis of lncRNAs

To identify lncRNAs targeting miRNAs, the starBase version 2. (https://rnasysu.com/encori/) and DIANA-lncBase version 3. (https://diana.e-ce.uth.gr/lncbasev3) online database were used to construct lncRNA-miRNA pairs. These databases provide a user-friendly interface and comprehensive resource for experimentally validated lncRNA-miRNA interaction networks [57–59]. Additionally, DIANA-lncBase version 3. was employed to predict miRNA targets of DElncRNA, obtained from the GSE42387 dataset, resulting in the construction of DElncRNA-miRNA pairs.

Enrichment analysis of predicted lncRNAs was performed using the LncSEA version 1.0 platform (https://bio.liclab.net/LncSEAv1/index.php). LncSEA provides a comprehensive and freely accessible platform for conducting enrichment analysis of lncRNAs [60]. The analysis encompassed disease, subcellular localization, exosome and cancer hallmark categories, aiming to reveal the biological function of lncRNAs. A p-value < 0.05 was considered statistically significant.

### LncRNA-miRNA-mRNA networks construction and analysis

The lncRNA-miRNA-mRNA network was constructed by assembling the miRNA-mRNA, lncRNA-miRNA, and DElncRNA-miRNA pairs. This integrated network was then visualized using Cytoscape software. To identify the key lncRNAs associated with OXP-resistance in CRC, two criteria were employed. First, the degree score of each lncRNA node in the lncRNA-miRNA-mRNA network was calculated using the CytoNCA plug-in in Cytoscape. The top five lncRNAs with the highest scores were then selected.

Second, the association between expression of these top five lncRNAs and the OS rate of CRC patients were assessed using the Kaplan-Meier plotter (KM plotter) online database (https://kmplot.com/analysis/), with log-rank p-value < 0.05 considered statistically significant. The KM plotter database provides both data sources and OS information sourced from the Cancer Genome Atlas (TCGA) database [61, 62].

To explore the potential pathways involving each identified key lncRNA, DEGs associated with candidate lncRNAs were enriched using the clusterProfiler package, with a focus on KEGG pathways provided by the SRplot bioinformatics tool (http://bioinformatics.com.cn/). SRplot is an online tool integrating clusterProfiler in R packages, facilitating data analysis and visualization. Enrichment results with a p-value < 0.05 were considered statistically significant.

### Cell culture

The HCT116 human colorectal carcinoma cell line was purchased from the Pasteur Institute of Iran, Tehran. Cells were cultured in DMEM/F12 medium (Biowest, France), containing 10% FBS (Biowest, France) and 1% penicillin/streptomycin (Biowest, France). They were maintained under controlled conditions at 37°C with 5% CO_2_.

### Establishment of OXP-resistance HCT116 sub-cell line

To establish the HCT116/OXR sub-cell line, parental HCT116 cells were exposed to gradually increasing concentrations of OXP (Sigma, USA), as described previously [8, 63]. The culture medium used to generate the OXP-resistant sub-cell line remained consistent with that of the parental HCT116 cells, comprising DMEM/F12, to prevent potential cellular shock during resistance development. In this procedure, the parental HCT116 cells were initially cultured in a drug-free medium for 24 h. Subsequently, during the logarithmic phase of cell growth, the medium was replaced with a culture medium containing an initial concentration of 1 μM OXP, which is lower than the 72 h IC_50_. After incubation for 72 h, the medium was removed, and the cells were maintained in drug-free medium for at least one week to allow for recovery. The rescued cells underwent passaging and were then re-exposed to the same concentration of the drug. This process was repeated several times until the cells exhibited consistent proliferation in the drug-containing medium. Once stable, the cells were treated with increasing concentrations of the drug (2.5, 5, 10, 20 and finally 30 μM) over a period of 10 months. This procedure was repeated for each concentration until consistent proliferation in the drug-containing medium was observed. OXP-resistance in cells was confirmed through cell viability assays, resistance index measurements, cell proliferation assay, cell apoptosis assays and cell cycle progression analysis. The HCT116/OXR sub-cell line was maintained in a medium containing 30 μM OXP to sustain resistance. Before further experiments, the cells were incubated in a drug-free medium for at least two weeks to allow for recovery.

#### Cell viability assay

HCT116 and HCT116/OXR cells were seeded in 96-well culture plates (8×10^3^ cells/well) and incubated overnight at 37°C. The culture medium was then replaced with fresh medium containing different concentrations of OXP (0.1–1000 μM). After incubation for 72 h, the medium was removed and 20 μl of MTT solution (5 mg/ml in PBS; Thiazolyl blue tetrazolium bromide, Life Biolab, Germany) was added to each well. The cells were incubated for an additional 3 hours at 37°C to allow MTT formazan crystal formation. The crystals were solubilized with 100 μl of DMSO, and the optical density (OD) was measured at 570 nm using an ELISA Plate Reader (Anthos, UK). Cell viability was calculated as a percentage relative to the untreated control cells. The resistance index was determined as the ratio between the IC_50_ values of HCT116/OXR cells and parental HCT116 cells [11]. This experiment was replicated three times to ensure consistency.

#### Cell proliferation assay

To assess the proliferative activity of the HCT116 and HCT116/OXR cells, 5×10⁴ cells/well were seeded in 24-well culture plates without any drugs. The cells were counted at 24 h intervals over a continuous period. The doubling time was calculated using the formula: Doubling Time = Duration (hours) × LN (2) / LN (final concentration / initial concentration). This process was replicated three times to ensure experimental consistency.

#### Cell apoptosis assay

Flow cytometry was conducted to analyze Annexin V-FITC/propidium iodide (PI) staining for assessing cell apoptosis. HCT116 and HCT116/OXR cells were seeded in 12-well culture plates at a density of 5×10⁴ cells/well and incubated overnight at 37°C. After 24 h, the culture medium was replaced with fresh medium containing 75 μM of OXP, and the cells were incubated for an additional 48 hours. The concentration of 75 μM was selected based on the IC50 value of the OXP-resistant sub-cell line, as it was required to elicit a strong apoptotic response within the 48hour timeframe. This higher concentration ensured a measurable apoptotic response while minimizing non-specific cytotoxic effects. Then, the cells were washed with PBS and trypsinized using trypsin-EDTA (Biowest, France), followed by neutralization with a complete medium. The cells were centrifuged at 1500 rpm for 5 minutes, and the cell pellets were resuspended in PBS. 100μl of Annexin V-Binding Buffer (BioLegend, UK), 5μl Annexin V-FITC (BioLegend, UK) and 10 μl PI were added to the pellet of cells. The mixture was incubated for 15 minutes at room temperature in the dark. After incubation, the labeled cells were diluted with 400 μl binding buffer and analyzed using BD FACS Calibur Flow Cytometer (BD Biosciences, San Jose, CA, USA). The data were analyzed using FlowJo software (version 7.6.1). The percentage of early and late apoptotic cells was determined to assess the apoptosis rate.

#### Cell cycle assay

Flow cytometry was conducted to assess the cell cycle profile. HCT116 and HCT116/OXR cells were seeded in 12-well culture plates (5×10^4^ cells/well) and incubated overnight at 37°C. After 24 h, the culture medium was replaced with a fresh medium containing 30 μM of OXP, and the cells were incubated for an additional 48 h. The concentration of 30 μM was chosen based on the maximum level at which the resistant cells could still grow. This concentration was adequate for observing subtle alterations in cell cycle distribution without causing excessive cell death, which could interfere with accurate measurements. Then, the cells were washed with PBS and trypsinized, followed by neutralization with a complete medium. The cells were collected and stained with 40 μl of PI, 10 μl of RNase (DNase-free) and 950 of μl PBS, and incubated for 30 minutes in the dark at room temperature. The cell cycle phase distribution was then analyzed using BD FACS Calibur Flow Cytometer (BD Biosciences, San Jose, CA, USA). The data were analyzed using FlowJo software (version 7.6.1).

### Exosomes isolation and characterization

To isolate the exosomes, HCT116 and HCT116/OXR cells were cultured in an FBS-free medium for 48 h. The conditioned medium supernatants were collected and subjected to stepwise centrifugation at 4°C to remove cells, dead cells and cell debris. First, the supernatants were centrifuged at 500× g for 20 minutes. Then, the remaining supernatants were centrifuged at 2500 × g for 20 minutes. Finally, the remaining supernatants were centrifuged at 14000 × g for 45 minutes. The resulting supernatants were concentrated using an ultrafiltration Amicon Ultra-15 100 kD device (Merck Millipore, Germany) at 4000 × g. Exosomes were isolated from the concentrated supernatant using an isolation kit (Cibzist, Iran) following the manufacturer’s protocol. The isolated exosomes were characterized based on size distribution, morphology, and the presence of exosomal markers to confirm successful isolation.

#### Dynamic light scattering (DLS)

The size distribution of exosomes was measured using the DLS method with a HORIBA Scientific Nanoparticle Analyzer (SZ-100), following the manufacturer’s guidelines.

#### Transmission electron microscopy (TEM)

The morphology of exosomes was evaluated using TEM. Briefly, a drop (20 μL) of isolated exosomes was dripped onto a 300-mesh carbon-coated TEM grid (EMS, USA) for 2 min. The excess liquid was then removed using filter paper. Negative staining was performed by applying a drop (20 μL) of 2% uranyl acetate for 1 minute. The excess liquid was again removed with filter paper, and the grid was allowed to air dry. The prepared grids were examined using a TEM (Zeiss, EFM10C) operating at an accelerating voltage of 100kV for image acquisition and analysis.

#### Western blot analysis

The presence of the exosomal markers CD9 and CD63 was determined by Western blot analysis. First, exosomes were lysed using RIPA lysis buffer. The concentration of proteins was assessed using the BCA Protein Quantification Kit (DNAbiotech, Iran). After denaturation at 95°C for 10 minutes, the proteins were separated by electrophoresis on a 10% SDS-polyacrylamide gel and transferred onto a polyvinylidene difluoride (PVDF) membrane (Roche, Mannheim Germany). The membrane was blocked with 2% skimmed milk (Sigma, Germany) in 1X Tris-buffered saline with 0.1% Tween-20 (TBST) for a one hour at room temperature. The blot was then incubated with specified primary antibodies against CD63 (#sc-5275, Santa Cruz Biotechnology, USA) and CD9 (#sc-13118, Santa Cruz Biotechnology, USA) overnight at 4°C. Subsequently, the membrane was washed with TBST buffer and incubated with m-IgGκBP-HRP secondary antibody (#sc-516102, Santa Cruz Biotechnology, USA) for one hour at room temperature. After another wash with TBST buffer, the signal was detected using chemiluminescence assay (ECL advanced reagents kit, Amersham, USA).

### Validation of predicted key lncRNAs

Cellular and exosomal RNAs were extracted from the HCT116 cell line and HCT116/OXR sub-cell line using One Step-RNA Reagent (Bio Basic, Germany) according to the manufacturer’s protocol. RNA concentration and quantity were measured using a spectrophotometer (Biotek, USA), with A260/280 ratios between 1.8 and 2.0. Subsequently, 1 μg of extracted RNA was reverse-transcribed into cDNA using the AddScript cDNA Synthesis Kit (Addbio, Korea), following the manufacturer’s instructions. Real-Time qRT-PCR was performed in triplicate on the StepOnePlus Real-Time PCR System (Thermo Fisher Scientific, Germany) using 1 μl of cDNA as a template and RealQ Plus 2x Master Mix Green High ROX^™^ (Ampliqon, Denmark). The thermal cycling protocol included an initial denaturation at 95°C for 15 minutes, followed by 40 cycles of denaturation at 95°C for 20 seconds and annealing/extension at 72°C for 30 seconds. Relative gene expression levels were normalized to GAPDH and calculated using the 2^-ΔΔCt^ method. The primers used for qRT-PCR are listed in Table 1.

**Table 1:** Primers for qRT-PCR of the three identified key lncRNAs.

Gene	Forward Sequences (5’ →3’)	Reverse Sequences (5’ →3’)
*NEAT1*	TGGCTAGCTCAGGGCTTCAG	TCTCCTTGCCAAGCTTCCTTC
*OIP5-AS1*	TGCGAAGATGGCGGAGTAAG	TAGTTCCTCTCCTCTGGCCG
*MALAT1*	GACGAGTTGTGCTGCTATCTT	GATTCTGTGTTATGCCTGGTTAG
*GAPDH*	GAAGGTGAAGGTCGGAGTCAACC	AGAGTTAAAAGCAGCCCTGGT

*NEAT1*: nuclear paraspeckle assembly transcript 1; *OIP5-AS1*: Opa-interacting protein 5 antisense RNA 1; *MALAT1*: Metastasis Associated Lung Adenocarcinoma Transcript 1; *GAPDH*: glyceraldehyde 3-phosphate dehydrogenase; Forward: Forward primer; Reverse: Reverse primer.

### Statistical analysis

The mean ± standard deviation (SD) of results from three independent experiments was reported. Statistical analysis was conducted using GraphPad Prism 8 (GraphPad Software, LLC). A Student’s t-test was employed to compare differences between the two groups. A p-value < 0.05 was considered as statistically significant.

## Results

### Identification of DEGs and DElncRNAs

**Fig 1:**
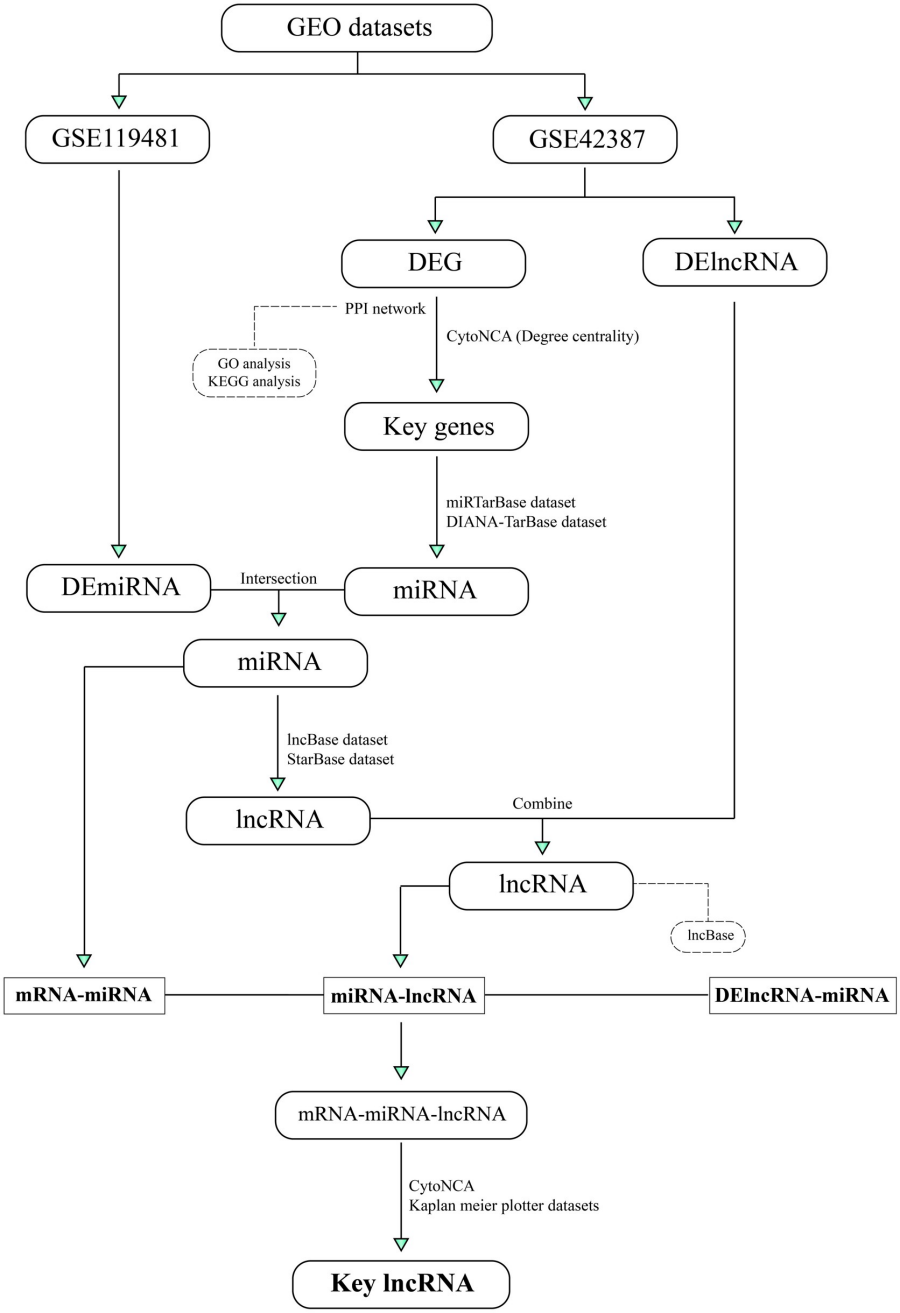
Bioinformatics analysis flowchart. This flowchart illustrates the stepwise bioinformatics procedure utilized for identifying key lncRNAs associated with OXP-resistance in CRC. OXP, Oxaliplatin; CRC, Colorectal Cancer.

The bioinformatics analysis flowchart conducted in the current study is shown in Fig 1.

**Fig 2:**
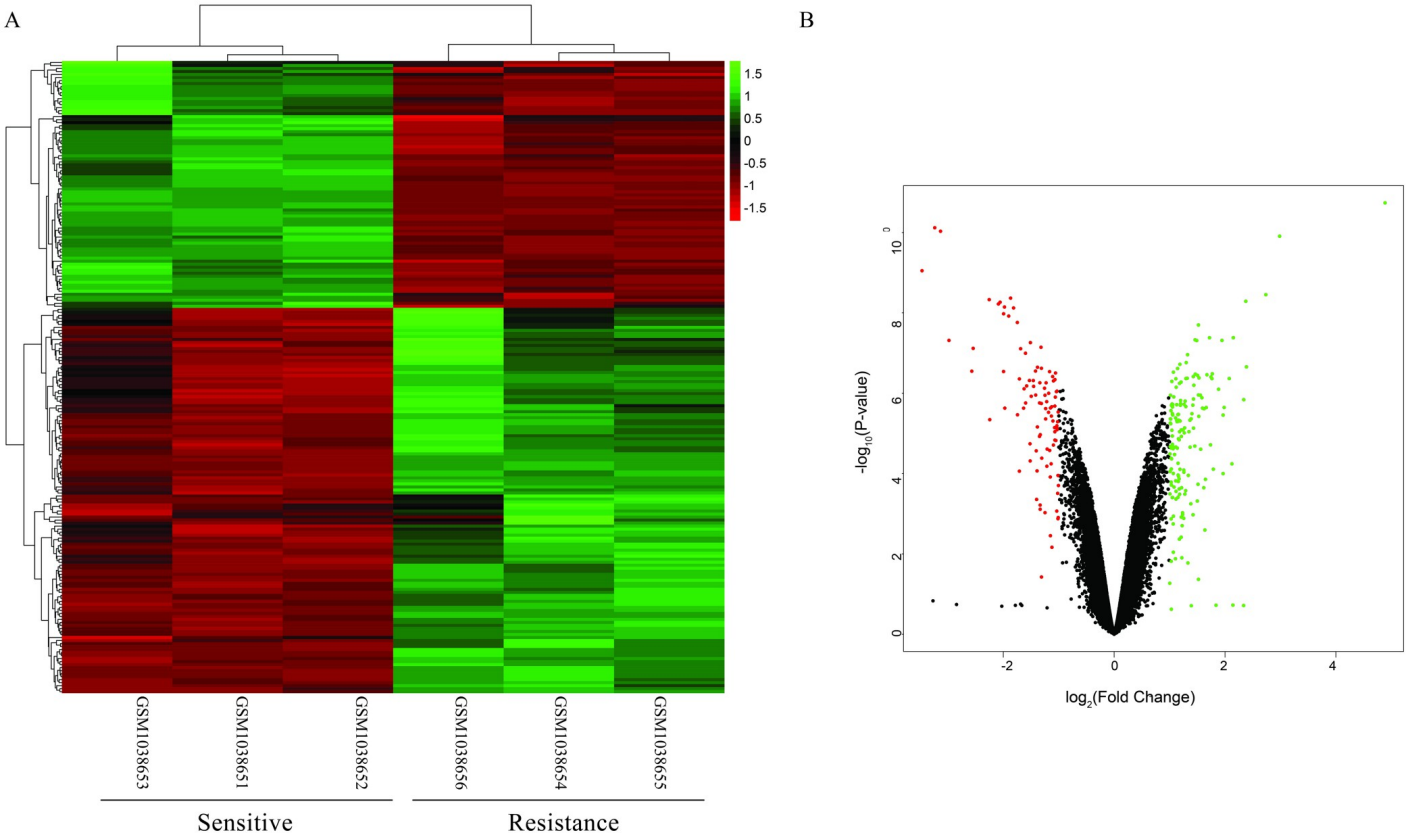
Heatmaps and volcano plots illustrating gene expression patterns associated with OXP-resistance in HCT116 cells, identified from the GSE42387 dataset (|log2 fold change (FC)| > 1, p-value < 0.05). (A) Heatmap displaying gene expression patterns, with sample names on the horizontal axis and fold-change on the vertical axis. Up-regulated genes are indicated in green, while down-regulated genes are shown in red. (B) Volcano Plot demonstrating the association between alterations in gene expression fold change and their statistical significance. Green dots indicate up-regulated genes, while red dots represent down-regulated genes. Among the genes, *HCLS1*, *EHF*, *KIRREL2*, *FGF9*, *ATG4A*, *CALB2*, *COL13A1*, *PMEPA1*, *BST2*, and AKR1C3 were identified as the top 10 up-regulated genes, whereas *IAH1*, *SUSD2*, *S100A4*, *FMR1*, *KRT23*, *HDGF*, *ZNF266*, *STXBP6*, *CYB5B*, and *GNE* were the top 10 down-regulated genes. Additionally, the up-regulated lncRNAs *LINP1*, *LINC00326*, *LINC00707*, *LINC00992*, and *CCDC144NL-AS1* were identified, while *TMEM132D-AS1*, *SFTA1P*, *RARA-AS1*, and *LINC01405* were found to be down regulated. OXP, Oxaliplatin.

Analysis of the GSE42387 dataset identified a total of 210 DEGs, including 128 up- and 82 down-regulated genes, as well as 9 DElncRNAs, comprising 5 up- and 4 down-regulated lncRNAs, associated with OXP-resistance in CRC, following the specified cutoff criteria. A heatmap and a volcano plot were generated to visualize the expression patterns of these genes (Fig 2 and S1 Table).

### Identification of OXP-resistance -associated central genes in the PPI network and functional enrichment analysis of DEGs

**Fig 3:**
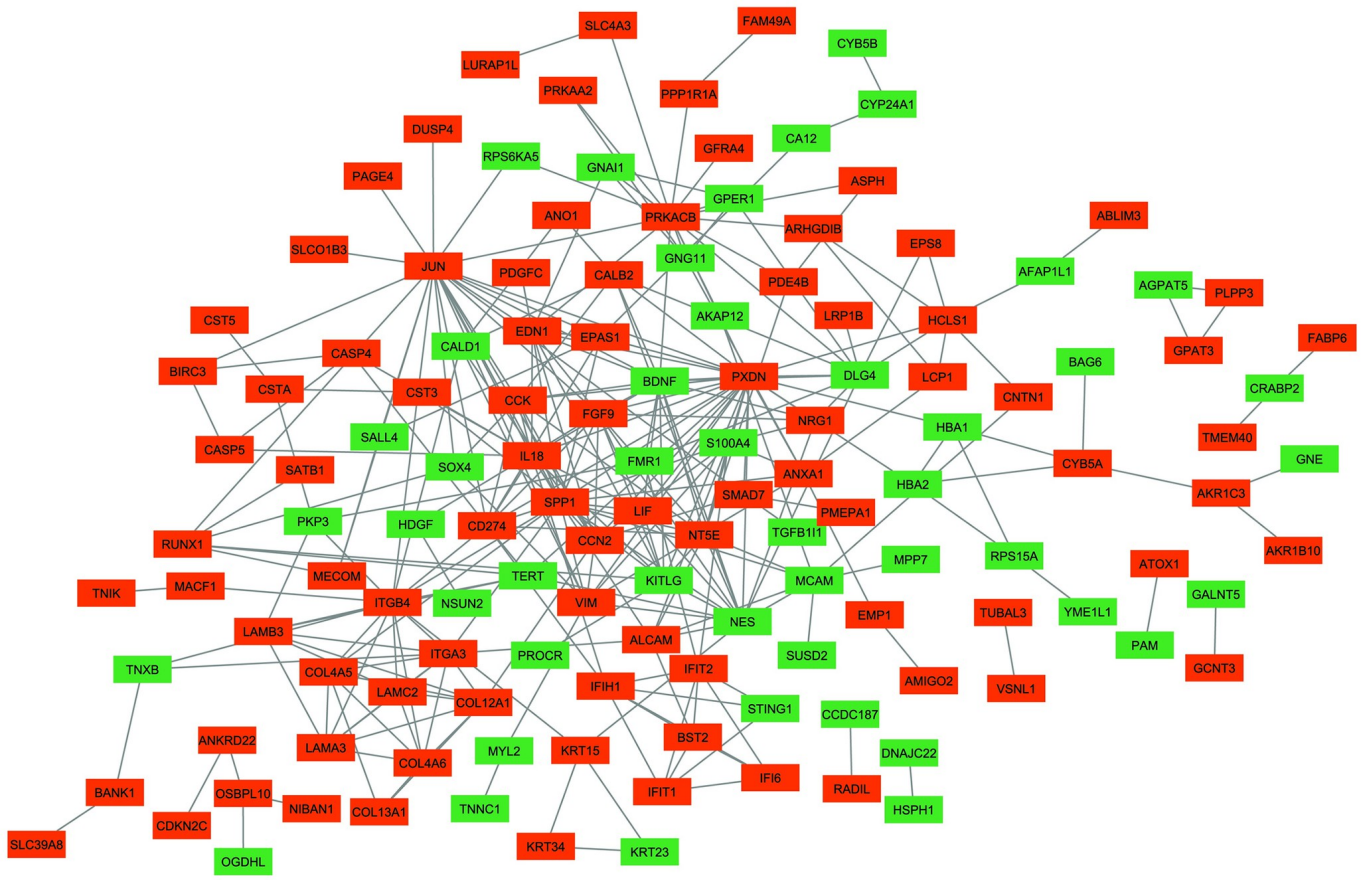
The PPI network of DEGS associated with OXP-resistance. The PPI network consists of 133 nodes (genes) and 297 edges (interactions). Orange rectangles display up-regulated genes, while green rectangles display down-regulated genes. DEG, Differentially expressed gene; PPI, Protein-protein interaction; OXP, Oxaliplatin.

The PPI network for OXP-resistance -associated DEGs were constructed. In total, 133 genes, including 87 up- and 46 down regulated, were involved in the network (Fig 3).

**Fig 4:**
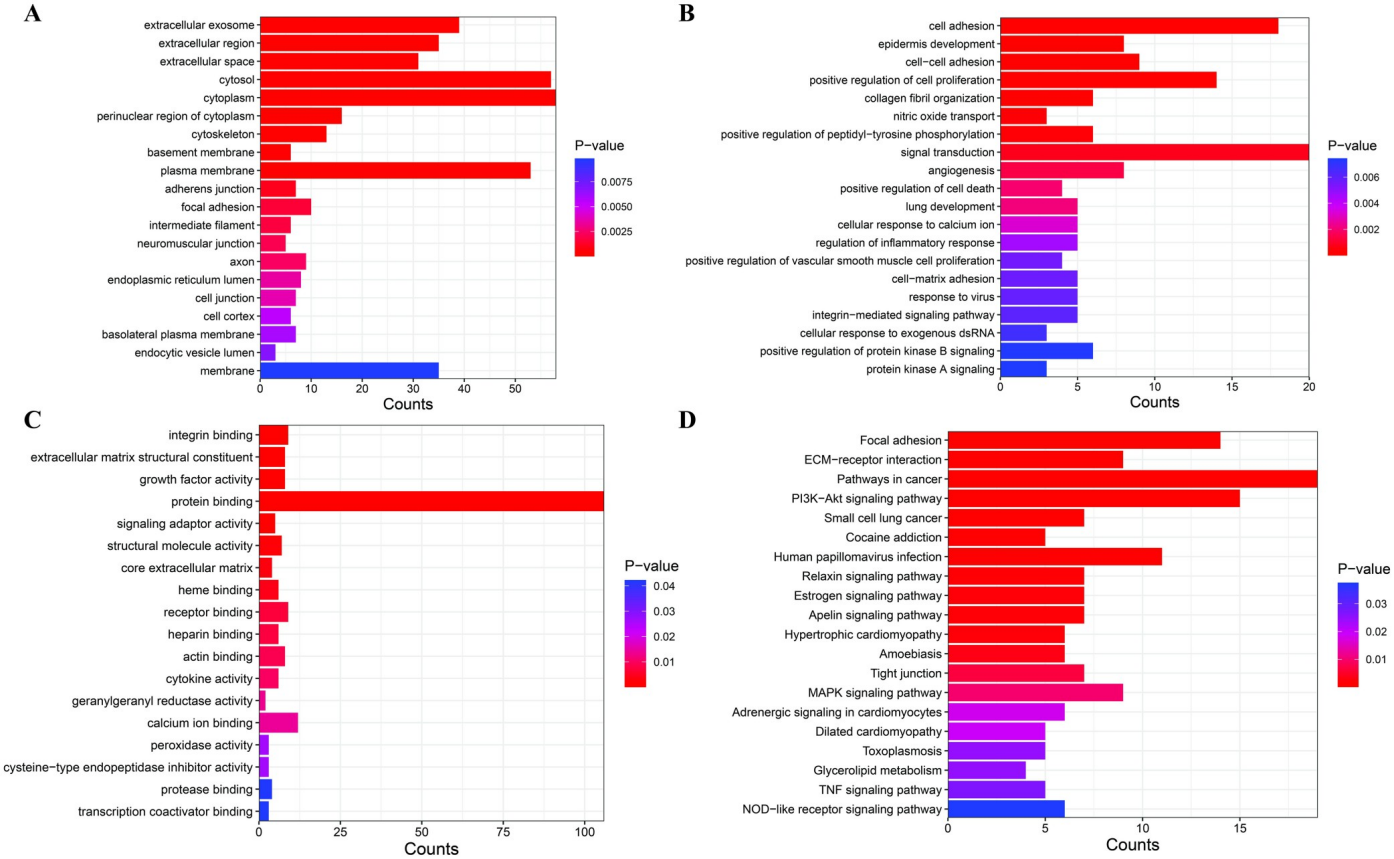
Functional enrichment analysis of genes in the PPI network. (A–C) Top 20 enriched GO Terms for CC, BP, and MF, respectively (D) Top 20 Significant KEGG pathway terms (p-value < 0.05). GO, Gene Ontology; CC, Cellular Component; BP, Biological Process; MF, Molecular Function; KEGG, Kyoto Encyclopedia of Genes and Genomes.

Enrichment analysis was conducted to investigate the functions and pathways of these genes. Fig 4 displays GO terms, including biological processes (BP), cellular components (CC), and molecular functions (MF), along with the KEGG pathways, ranked by their p-values (from low to high). Detailed results are presented in S2 Table. The visualizations were generated using the freely accessible online SRplot bioinformatics web server. The CC term indicates that most of the genes were components of the “extracellular exosome”, “extracellular region”, “extracellular space”, “cytosol” and “cytoplasm” (Fig 4A). From the viewpoint of BP, the majority of the genes were involved in “cell adhesion”, “positive regulation of cell proliferation”, “collagen fibril organization”, “positive regulation of peptidyl-tyrosine phosphorylation”, “nitric oxide transport”, “signal transduction” and “angiogenesis” (Fig 4B). The MF terms indicate that most of the genes were involved in “integrin binding”, “extracellular matrix structural constituent”, “growth factor activity”, “protein binding” and “signaling adaptor activity” (Fig 4C). The KEGG pathway analysis showed significant enrichment of these genes across 22 signaling pathways. The top-ranked pathways include “Focal adhesion”, “ECM-receptor interaction”, “Pathways in cancer” and “PI3K/Akt signaling pathway” and “MAPK signaling pathway” (Fig 4D). These pathways are known to regulate cellular processes such as growth, proliferation, survival, apoptosis, migration, and differentiation. Their significant enrichment underscores their pivotal role in modulating cellular processes relevant to CRC progression and chemotherapy resistance [43, 64–69]. In summary, the enrichment results suggest that these genes play significant roles in cancer development.

In the following analysis, the top 10 ranked genes in the network were identified using degree centrality. These genes, which exhibited the highest node degrees, include *PXDN*, *SPP1*, *FGF9*, *NES*, *JUN*, *BDNF*, *ITGB4*, *PRKACB*, *IL-18*, and *CD274* that considered as central genes and used for future analysis (Table 2).

**Table 2:** The top 10 genes in the PPI networks.

Gene symbol	Official gene name	Degree
*PXDN*	Peroxidasin	26
*JUN*	Jun proto-oncogene, AP-1 transcription factor subunit	23
*SPP1*	Secreted Phosphoprotein 1	22
*PRKACB*	Protein Kinase cAMP-Activated Catalytic Subunit Beta	17
*ITGB4*	Integrin Subunit Beta 4	15
*BDNF*	Brain-Derived Neurotrophic Factor	15
*NES*	Nestin	15
*FGF9*	Fibroblast Growth Factor 9	13
*IL18*	Interleukin 18	13
*CD274*	CD274 Molecule	12

### Predicted miRNAs

The upstream miRNAs targeting the top 10 genes were predicted by combining data from TarBase and miRTarBase, resulting in the identification of 520 experimentally validated miRNAs (S3 Table).

**Fig 5:**
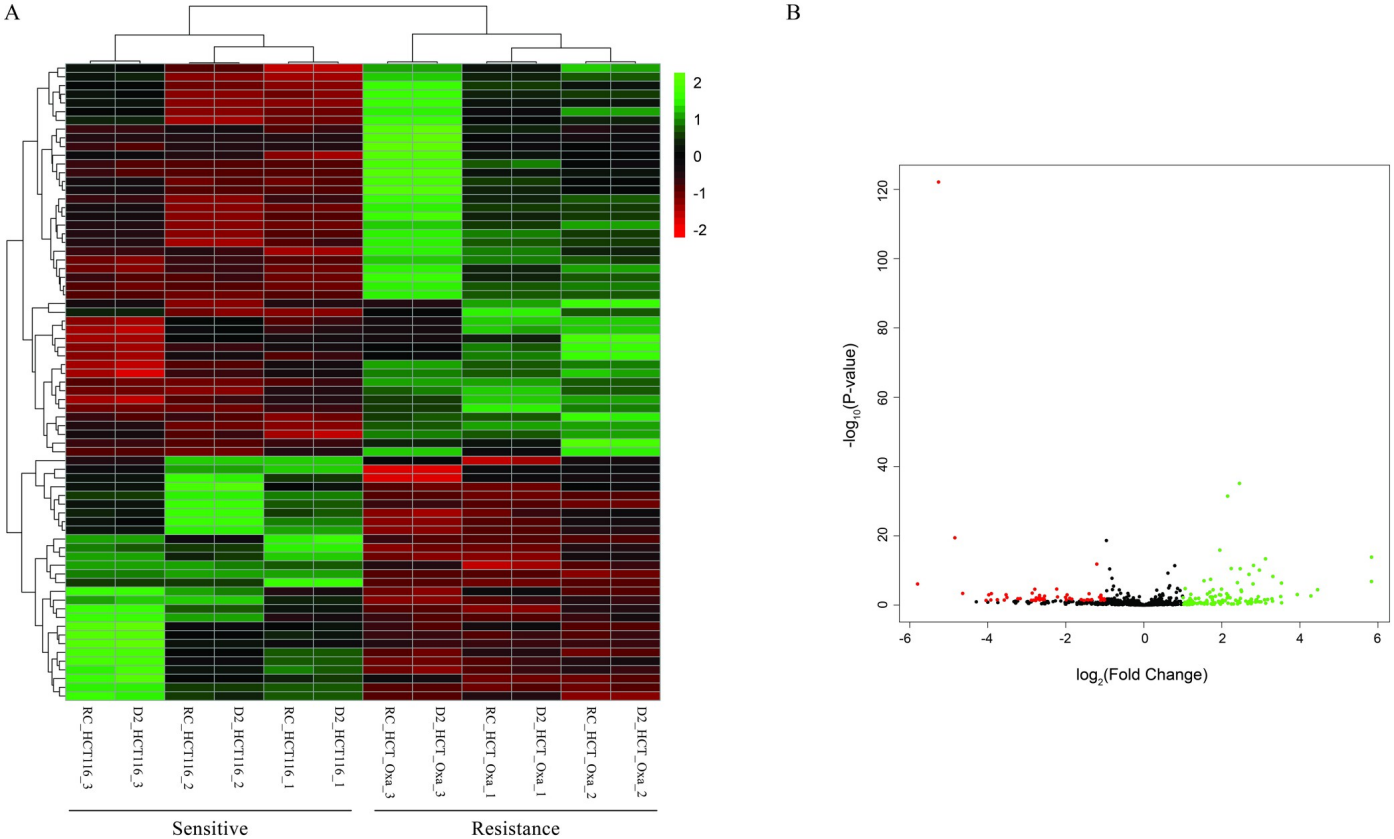
Heatmaps and volcano plots illustrating miRNA expression patterns associated with OXP-resistance in HCT116 cells, identified from the GSE119481 dataset (|log2 fold change (FC)| > 0.6, p-value < 0.05). (A) Heatmap displaying miRNA expression patterns, with sample names on the horizontal axis and fold change on the vertical axis. Up-regulated miRNA are presented in green, while down-regulated miRNA are in red. (B) Volcano Plot demonstrating the association between alterations in expression fold change and their statistical significance. Green dots indicate up-regulated miRNA, while red dots represent down-regulated miRNA. OXP, Oxaliplatin.

Additionally, analysis of the GSE119481 dataset identified 73 DEmiRNAs, comprising 28 upregulated and 45 downregulated miRNAs associated with OXP-resistance in CRC, following the specified cutoff criteria. Subsequently, a heatmap and a volcano plot were generated to visualize the expression patterns of these DEmiRNAs (Fig 5 and S4 Table).

**Fig 6:**
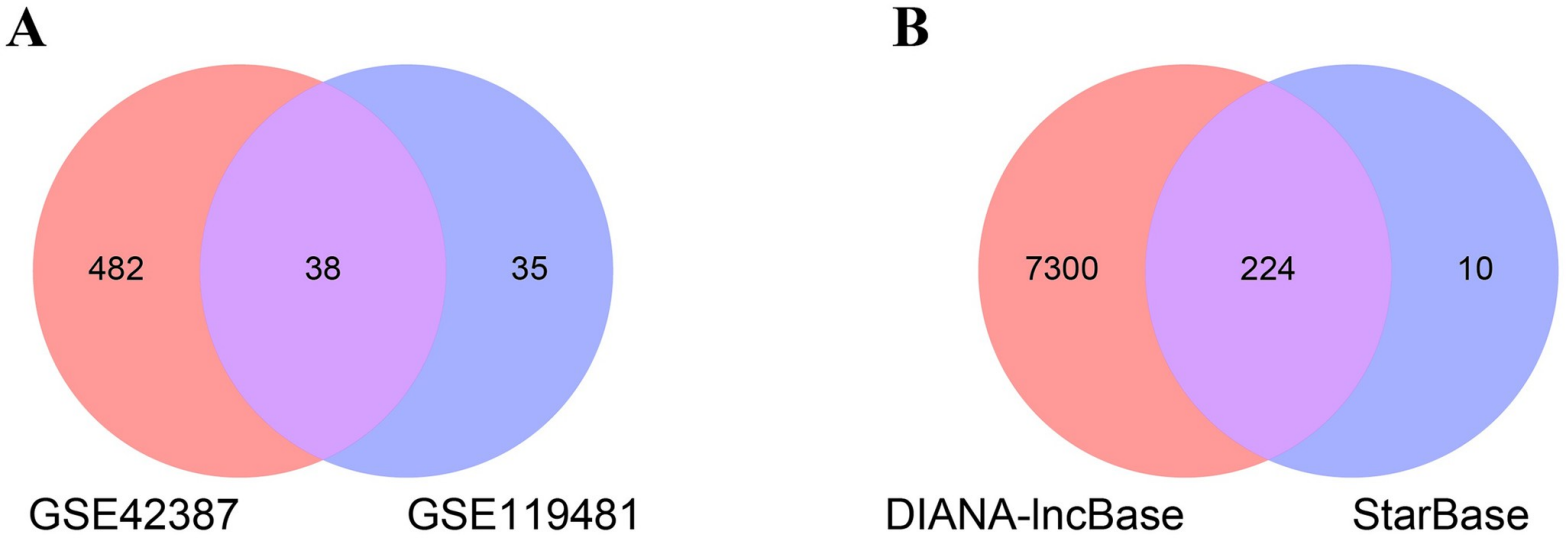
The Venn diagram of overlapped data. (A) The intersection between the predicted miRNAs and the DEmiRNAs to identify common miRNAs. (B) The intersection between the predicted lncRNAs from the StarBase and DIANA-lncBase databases to identify common lncRNAs. DEmiRNA, Differentially Expressed miRNAs.

After performing an intersection analysis between the predicted miRNAs and the DEmiRNAs using a Venn diagram generated by the SRplot bioinformatics web server, 38 common miRNAs were identified. Subsequently, miRNA-mRNA pairs were constructed (Fig 6A and S5 Table).

### Predicted lncRNAs and enrichment analysis

Using StarBase and DIANA-lncBase, 224 lncRNAs targeting 36 out of 38 common miRNAs were predicted, and lncRNA-miRNA pairs were constructed (Fig 6B and S6 Table). Additionally, 10 miRNAs were predicted for DElncRNAs which obtained from the GSE42387 dataset (S6 Table), followed by the construction of DElncRNA-miRNA pairs.

The four relevant categories—disease, subcellular location, exosome, and cancer hallmark of the lncSEA platform were explored for the predicted lncRNAs. Detailed results are provided in S7 Table. Overall, in the disease category, most of the predicted lncRNAs were primarily enriched in CRC. The cytoplasm, nucleus, and exosome were the main locations for these lncRNAs in the subcellular locations category. Previous studies have supported the cooperative relationships between lncRNAs and hallmark of cancer [70, 71]. In the cancer hallmark category, the predicted lncRNAs were significantly enriched in processes such as proliferation, prognosis, invasion, apoptosis, migration, metastasis, and epithelial-mesenchymal transition (EMT).

### Identification of OXP-resistance -associated lncRNAs in constructed lncRNA-miRNA-mRNA networks

**Fig 7:**
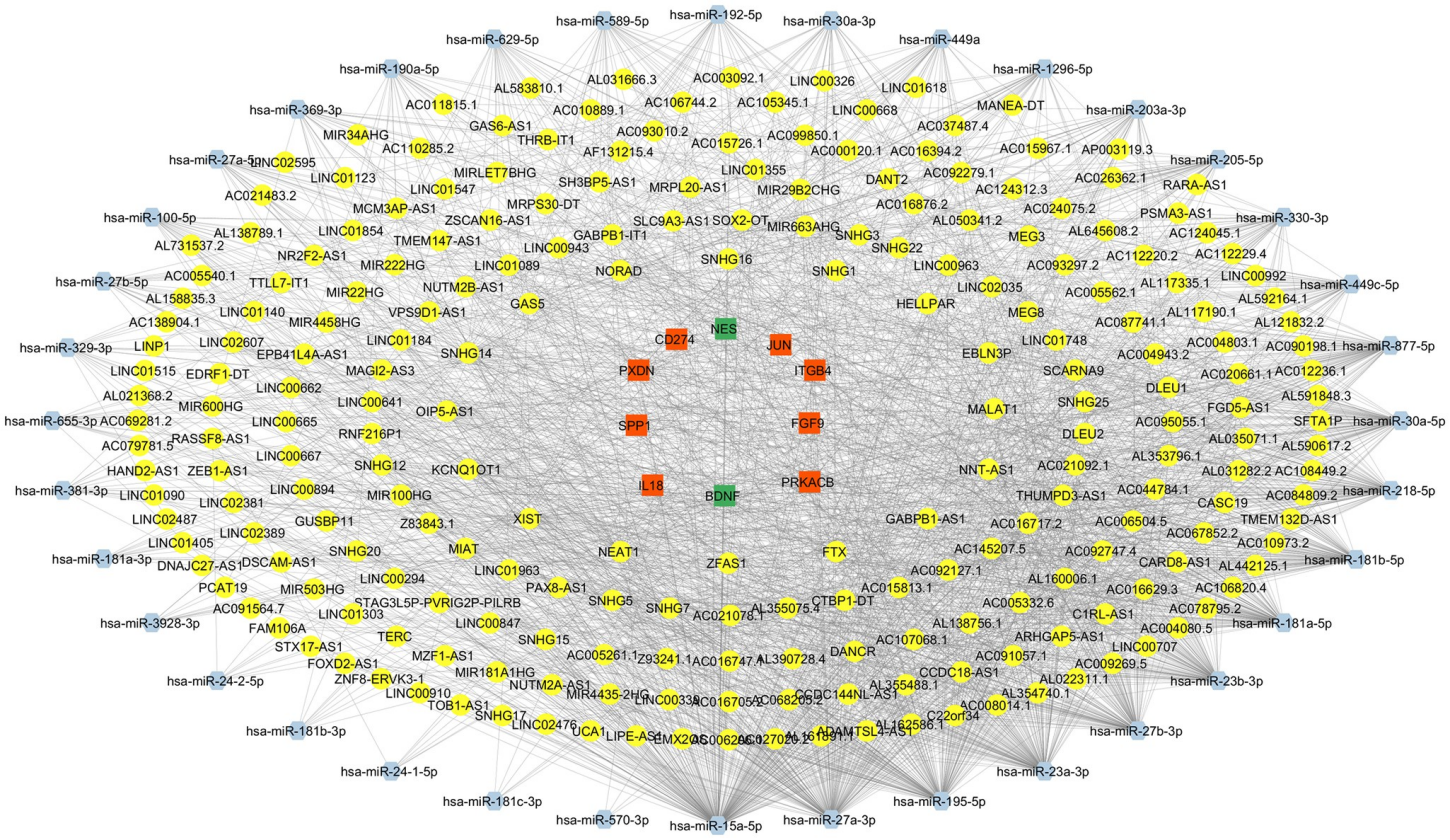
The lncRNA-miRNA-mRNA networks. This network contains 278 nodes and 2012 edges. Orange and green rectangles display up- and down-regulated genes, respectively; yellow ellipses represent lncRNAs, and blue hexagons represent miRNAs.

To screen for the key lncRNAs that regulate OXP-resistance -associated genes in CRC, the lncRNA-miRNA-mRNA network was constructed by assembling predicted miRNA-mRNA, lncRNA-miRNA, and DElncRNA-miRNA pairs (Fig 7). The degree centrality analysis of each lncRNA node highlighted that the top five lncRNAs—*NEAT1*, *MALAT1*, *XIST*, *OIP5-AS1*, and *SNHG14*—exhibited the highest node scores in the network. These lncRNAs were considered involved in OXP-resistance and were selected for further analysis.

### The prognostic impact of selected lncRNA expression on overall survival in CRC patients: Insights from the KM plotter platform

**Fig 8:**
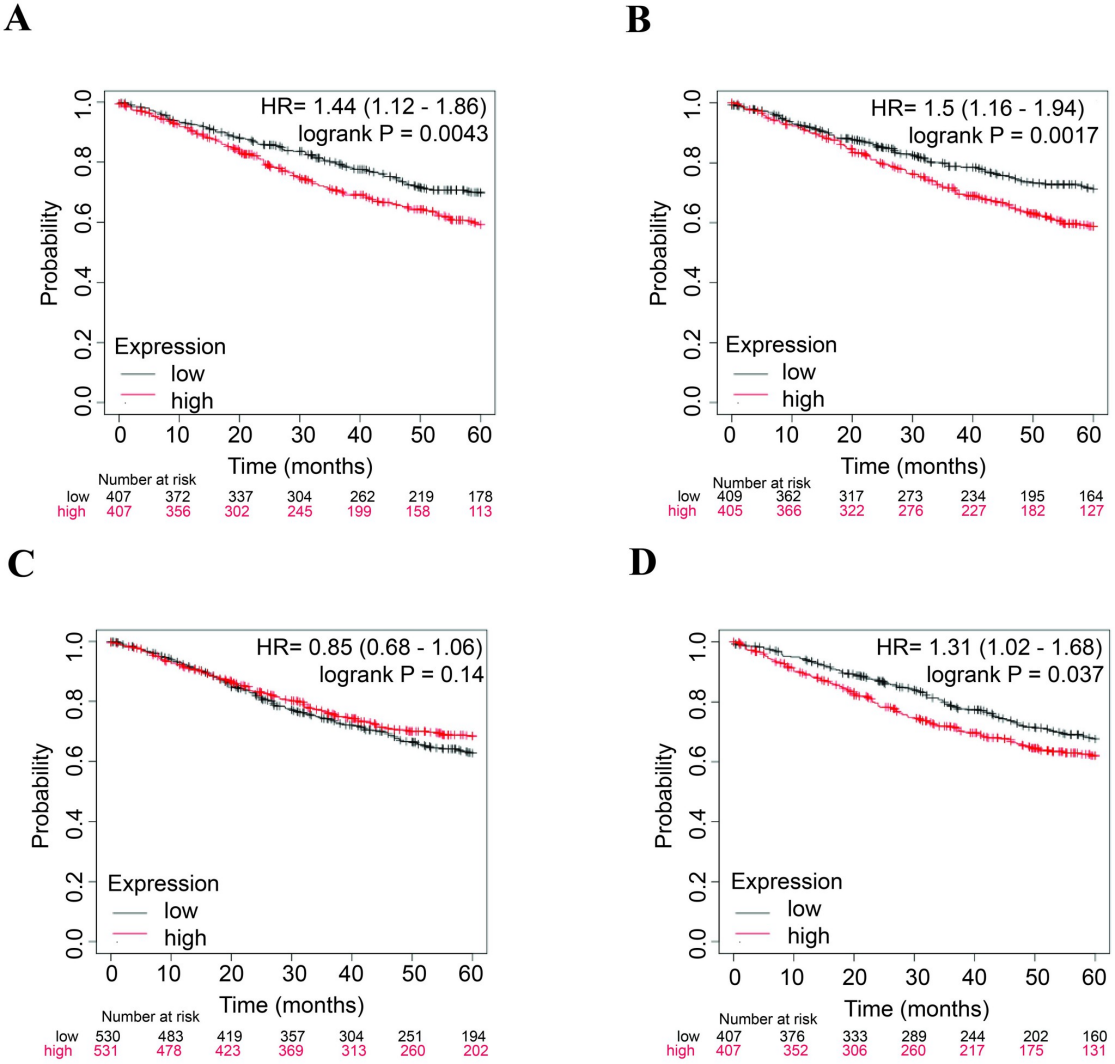
Kaplan–Meier overall survival curves for key lncRNAs in CRC patients. The horizontal axis of the graph shows the overall survival time in years, while the vertical axis indicates the corresponding survival probability. (A) *NEAT1*, (B) *MALAT1*, (C) *XIST* and (D) *OIP5-AS1*.

The potential association between the expression levels of each selected lncRNA and the OS of CRC patients was assessed using an available online database. The results indicated that high expression levels of lncRNAs *NEAT1*, *MALAT1*, and *OIP5-AS1* were significantly associated with reduced OS in CRC patients. However, *XIST* was not found to be associated with OS in CRC. Additionally, no data was available for *SNHG14* (Fig 8).

**Fig 9:**
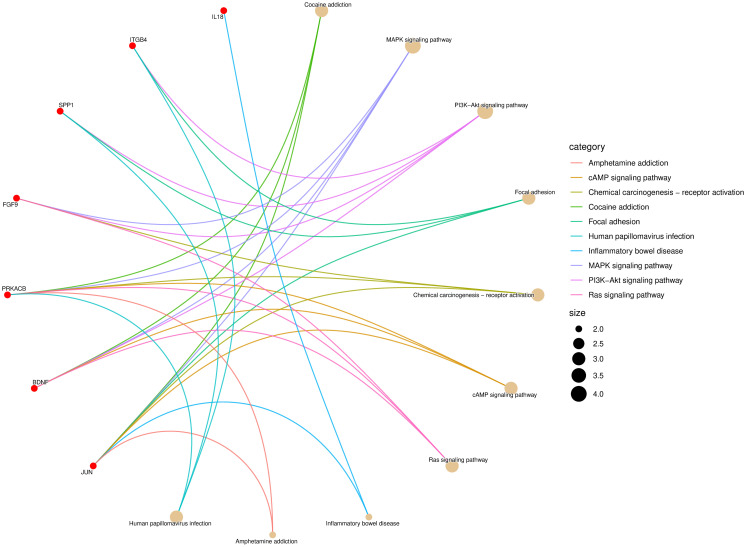
The pathway enrichment analysis for DEGs associated with *NEAT1*, *MALAT1*, and *OIP5-AS1*. This pathway performed using ClusterProfiler.

Finally, the lncRNAs *NEAT1*, *MALAT1*, and *OIP5-AS1*, which had high degree scores in the lncRNA-miRNA-mRNA network and significant associations with the overall survival (OS) of CRC patients, were identified as key lncRNAs potentially associated with OXP-resistance in CRC. To elucidate the biological functions of the three lncRNAs, the genes associated with these lncRNAs were enriched using ClusterProfiler. The results revealed that genes connected to the lncRNAs were involved in pathways such as the MAPK signaling pathway, PI3K/Akt signaling pathway, focal adhesion, chemical carcinogenesis, cAMP signaling pathway, and Ras signaling pathway (Fig 9 and S8 Table).

### Comprehensive characterization of HCT116/OXR sub-cell line

**Fig 10:**
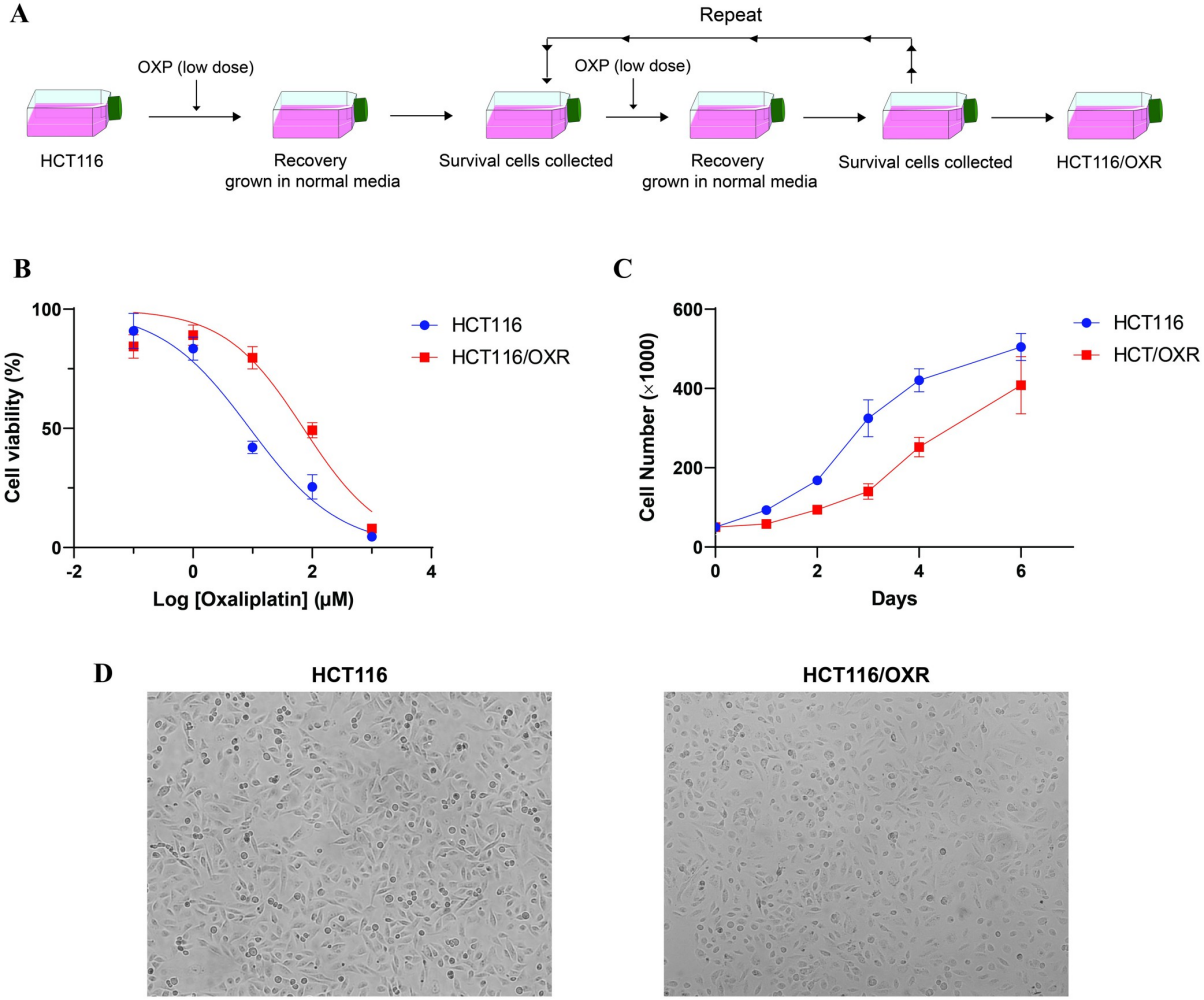
Establishment and characterization of HCT116/OXR sub-cell line. (A) The process of establishing OXP-resistant cells involved gradually exposing parental HCT116 cells to increasing concentrations of OXP over a period. (B) Cell viability dose-response curves for parental HCT116 and HCT116/OXR cells exposed to different concentrations of OXP (0.1–1000 μM). (C) Growth curve of parental HCT116 and HCT116/OXR cells. (D) Morphology of parental HCT116 and HCT116/OXR cells. OXP, Oxaliplatin; HCT116/OXR, Oxaliplatin-resistance HCT116.

HCT116/OXR cells were generated by exposing parental HCT116 cells to gradually increasing concentrations of OXP over a period of 10 months (Fig 10A). To ensure the establishment of acquired OXP-resistant cells, characterization assessments were performed. The cell viability assay showed that the IC_50_ value for parental HCT116 cells was 8.95 μM, whereas the HCT116/OXR sub-cell line exhibited an IC_50_ value of 71.48 μM (Fig 10B). The OXP-resistance index for HCT116/OXR was determined to be eight times higher than that of parental cells. Following a drug-free culture of HCT116/OXR cells for three weeks and an extended period, the drug-resistant cells consistently demonstrated stable IC_50_ and relative resistance values. The growth curve analysis showed a slower growth rate of HCT116/OXR cells compared to HCT116 cells (Fig 10C). The doubling time in the HCT116/OXR sub-cell line was notably extended, averaging approximately 47 h. This represents an increase of about 1.8-fold compared to the parental cells, which had a doubling time of 27 h. The microscopic observation of cells using inverted microscopy indicated morphological alterations in HCT116/OXR compared to parental cells. The morphology of HCT116/OXR cells exhibited a pebble-like epithelial phenotype, with cells appearing smaller and more rounded in shape (Fig 10D).

**Fig 11:**
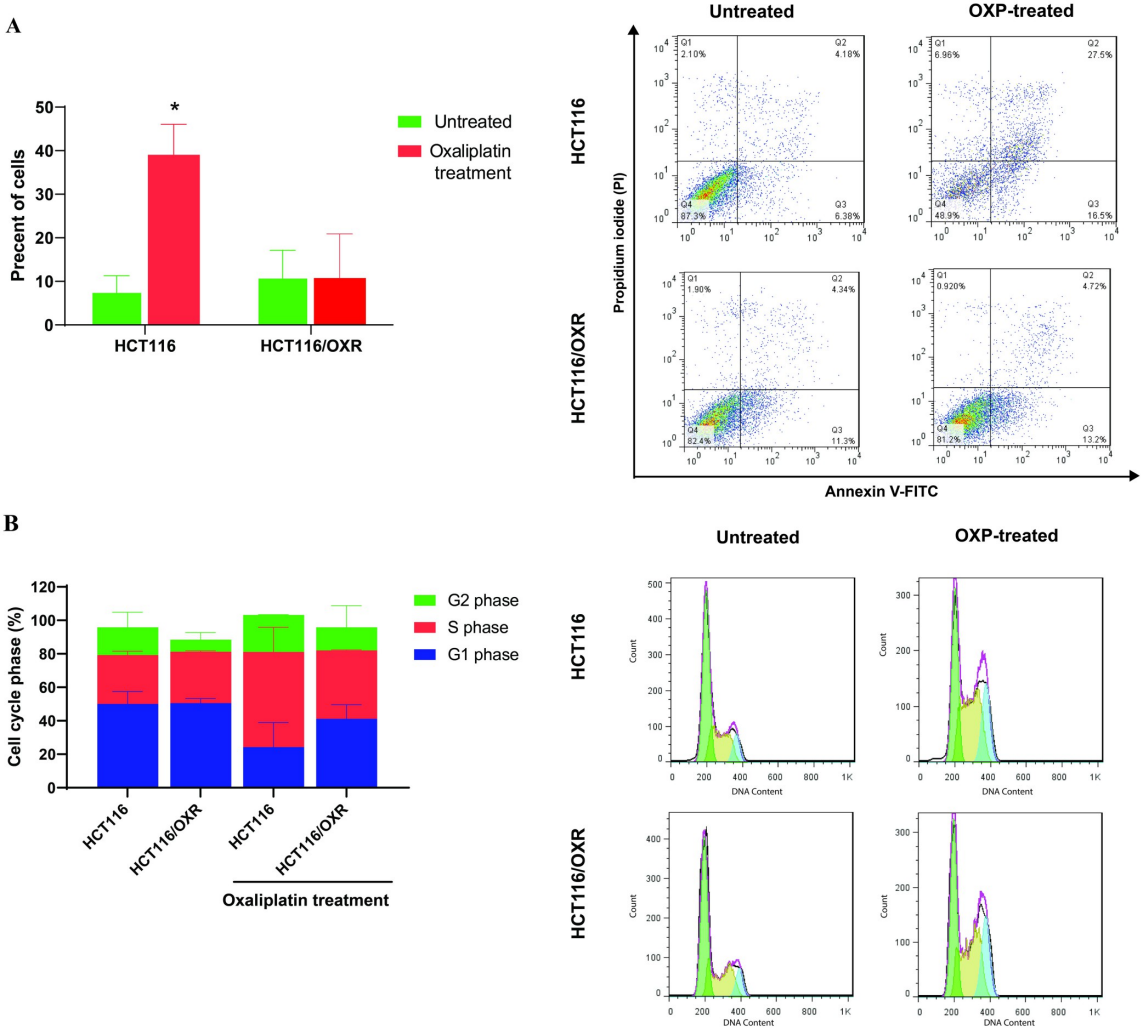
Apoptosis and cell cycle assay induced by OXP in parental HCT116 and HCT116/OXR cells using flow cytometry. (A) Left: Flow cytometric histograms of apoptosis induced by OXP in cells. Apoptosis was analyzed using Annexin V/PI staining. The diagram represents live cells (lower left), early apoptotic cells (lower right), late apoptotic cells (upper right), and necrotic cells (upper left). Right: Bar diagrams, representing the average counts of apoptotic cells obtained from at least two independent experiments. The percentage of early and late apoptotic cells determined the apoptosis rate. The data are presented as means ± SD (*p < 0.05). (B) Left: Flow cytometric histograms of cell cycle phase distribution under treatment with OXP in parental HCT116 and HCT116/OXR cells. Right: Stacked bar graphs showing the average counts of cells at cell cycle phase obtained from at least two independent experiments. OXP, Oxaliplatin; HCT116/OXR, Oxaliplatin-resistance HCT116.

The cell apoptosis assay demonstrated that OXP significantly induced apoptosis in parental HCT116 cells. However, under the same conditions, the OXP-resistant cells showed persistence despite OXP-induced apoptosis, with no significant alterations observed (Fig 11A). Analysis of cell cycle phase distribution showed that exposure to OXP led to significant S and G2/M phase arrest in parental HCT116 cells, whereas HCT116/OXR cells exhibited a delay in the G1 phase (Fig 11B). These results suggest that OXP-resistant cells overcome the cytotoxic effects of OXP by experiencing a prolonged G1 phase and reduced cell growth rate, which provides sufficient time for DNA damage repair. Moreover, by slowing down the cell cycle, they prevent the incorporation of drug metabolites into DNA. This strongly indicates the emergence of successful OXP-resistant sub-cell line s. In contrast, parental cells exhibit a reduced G1 phase and enter the S phase earlier. However, due to their inability to synthesize DNA and the presence of DNA damage, parental cells accumulate in the G2/M phase. Ultimately, because of the inability to repair DNA, apoptosis is initiated in parental cells.

### Comprehensive characterization of isolated exosomes

**Fig 12:**
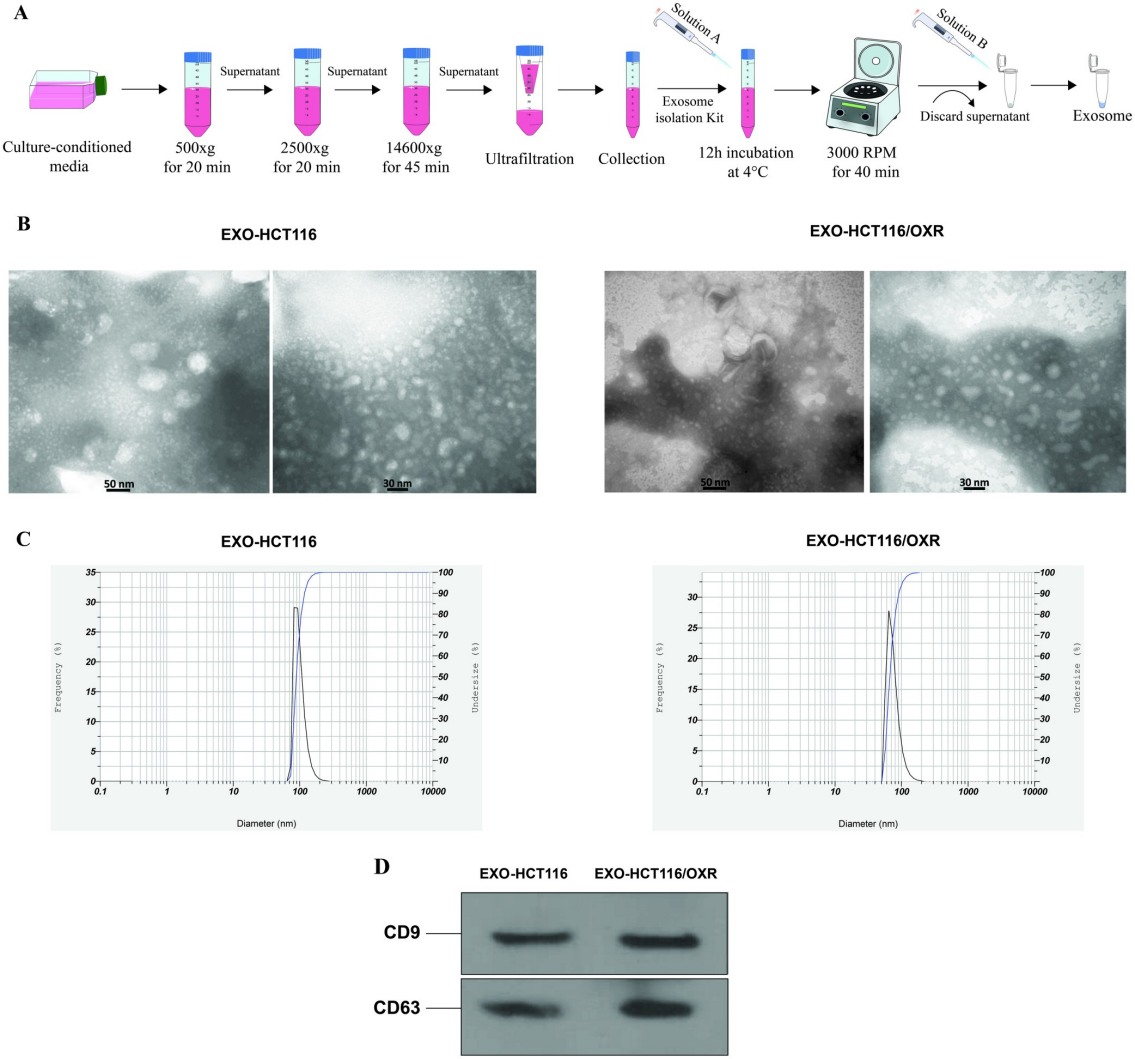
Characterization of exosomes derived from HCT116 and HCT116/OXR cells. (A) The process of exosome isolation, including stepwise centrifugation and the use of an isolation kit. (B) Representative TEM images showing the presence and morphology of exosomes. (C) Size distribution graphs of exosomes detected by DLS analysis. (D) Western blot analysis demonstrating the detection of exosomal markers CD9 and CD63 in isolated exosomes.

The exosomes were isolated from the parental HCT116 and HCT116/OXR cells according to the sequential steps indicated in Fig 12A and then characterized. TEM analysis showed the presence of exosomes with typical round shapes and bilayer membranes (Fig 12B). DLS revealed the expected sizes of exosomes, indicating that the average size was approximately 93.8 nm for HCT116 and 70.8 nm for HCT116/OXR (Fig 12C). Western blot analysis confirmed the enrichment of well-known exosome markers, CD9 and CD63, in the isolated exosomes (Fig 12D). These results demonstrate the successful isolation of exosomes.

### Association of cellular and exosomal *NEAT1*, *MALAT1*, and *OIP5-AS1* OXP-resistant in CRC

**Fig 13:**
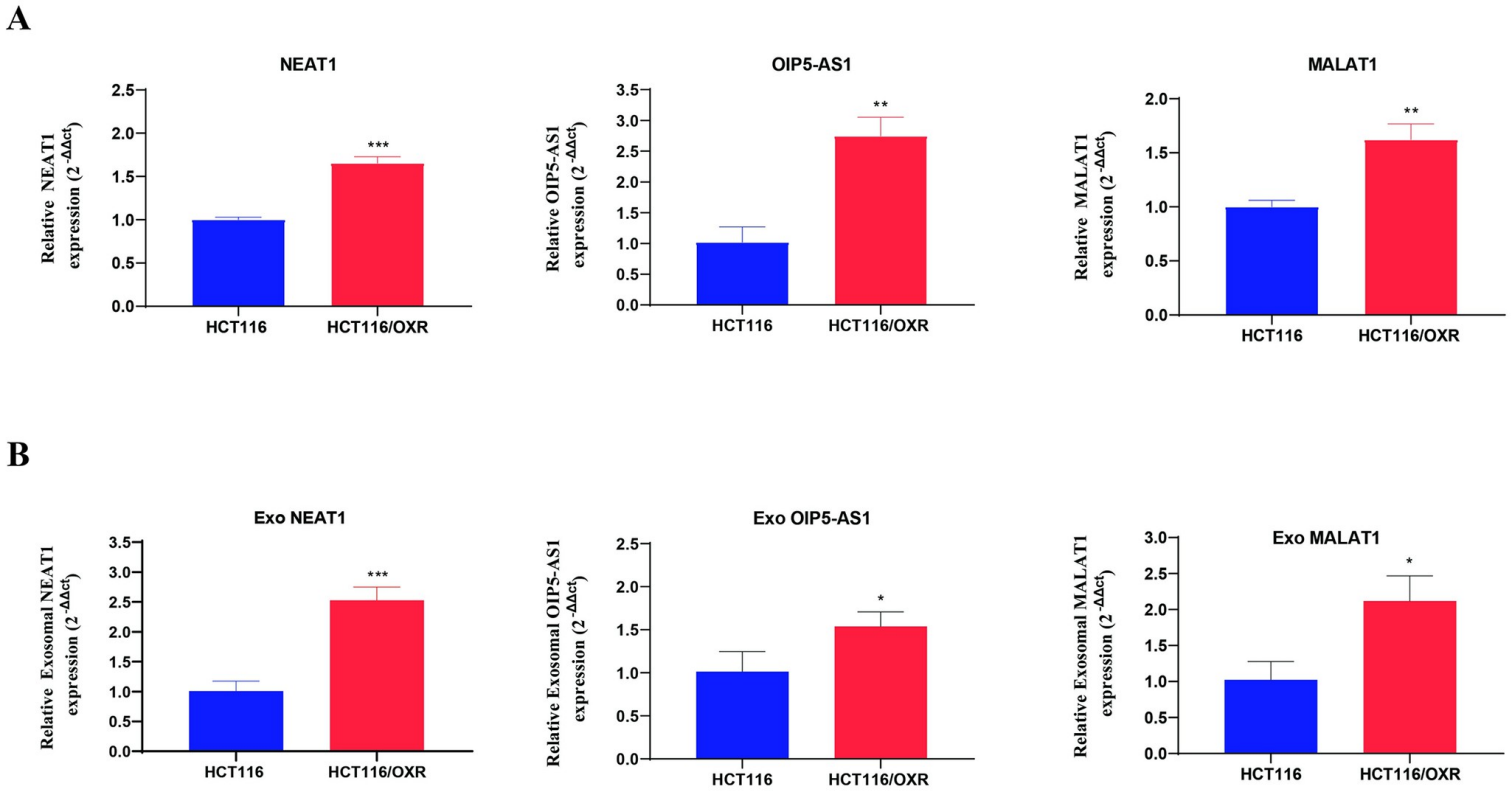
Relative expression levels of *NEAT1*, *OIP5-AS1* and *MALAT1* in parental HCT116 and HCT116/OXR cells and their isolated exosomes. (A) Bar diagrams present the expression levels of key lncRNAs in parental and OXP-resistance cells (B) Bar diagrams present the expression levels of key lncRNAs in isolated exosomes from parental and OXP-resistance cells. The data are presented as means ± SD. (* p < 0.05, **p < 0.01, and ***p < 0.001). OXP, Oxaliplatin; HCT116/OXR, Oxaliplatin-resistance HCT116; Exo, Exosome.

The experimental validation of the expression levels of the predicted key lncRNAs—*NEAT1*, *MALAT1*, and *OIP5-AS1*—in both HCT116/OXR and parental HCT116 cells was performed through qRT-PCR. The results revealed a significantly elevated expression level of all three lncRNAs in HCT116/OXR cells compared to parental cells (Fig 13A). This significant up-regulation shows a potential association between these lncRNAs and the observed phenotypic differences in the OXP-resistant cell line. As exosomes are well-recognized as critical mediators of intercellular communication, with their contents contributing to chemotherapy resistance [72], the exosomal form of these key lncRNAs in both parental and resistance-derived exosomes were also investigated. The results indicated that *NEAT1*, *OIP5-AS1*, and *MALAT1* were detectable in these isolated exosomes, with their expression levels being higher in the OXP-resistant group compared to the parental group (Fig 13B). Our data suggest that exosomal *NEAT1*, *OIP5-AS1*, and *MALAT1* could serve as promising diagnostic biomarkers for OXP-resistance in CRC.

## Discussion

Resistance to OXP is a cause of treatment failure and reduced survival rates in CRC patients, which remains a significant challenge in treatment [12]. Studies have identified several lncRNAs involved in OXP-resistance in CRC [14]. Considering the pivotal role of lncRNAs in the induction of chemotherapy resistance, investigating additional lncRNAs is crucial to enhancing treatment efficacy and overall patient survival. Furthermore, investigating the presence of lncRNAs in exosomes derived from OXP-resistant cells could reveal novel biomarkers for diagnosing resistance. Therefore, In the present study, we employed an in-silico approach to predict lncRNAs associated with OXP-resistance and then validated them using qRT-PCR in CRC cells and their isolated exosome.

Initially OXP-resistance associated DEGs from GSE42387 were obtained and a PPI network was constructed to identify the interconnected DEGs. Subsequently, *PXDN*, *SPP1*, *FGF9*, *NES*, *JUN*, *BDNF*, *ITGB4*, *PRKACB*, *IL-18*, and *CD274* were selected as central genes exhibiting the highest degree in the PPI network. Previous studies have reported that these genes play critical roles in cancer progression and chemotherapy resistance by regulating signaling pathways such as PI3K/Akt and Wnt/β-catenin, which are known to influence essential cellular processes including cell proliferation, survival and migration. For example, *SPP1*, encoding osteopontin, plays a crucial role in cell-matrix interactions and promotes tumor progression across various cancers, including CRC, prostate cancer, ovarian cancer, cervical cancer, and head and neck squamous cell carcinoma (HNSCC) [73, 74]. Elevated *SPP1* expression activates the integrin β1/FAK/Akt pathway, promoting cell proliferation, migration, and invasion [75]. Moreover, *SPP1* upregulation correlates with resistance to platinum-based drug and poorer survival rates in cancer patients [76–79]. Conversely, downregulation of *SPP1* expression promotes sensitivity to cisplatin by inhibiting the PI3K/Akt pathway in cervical cancer cells [80]. JUN, a component of the AP-1 (Activator Protein-1) transcription factor, promotes invasion and metastasis in various cancers when overexpressed. Zhang et al,. observed a significant correlation between *SPP1* and *JUN* expression and reduced survival rates in oral cancer patients [81]. Additionally, Liang et al., demonstrated that the upregulation of *LINC00174* induced by c-JUN contributes to proliferation and invasion in CRC [82]. FGF9, identified as a proto-oncogene, contributes to CRC progression and confers resistance to cisplatin by modulating the Wnt/β-catenin signaling pathway [83, 84]. Makondi et al. highlighted a positive correlation between *FGF9* and *PRKACB* expression in irinotecan-resistant CRC [85]. *NES*, frequently overexpressed in cancer cells, correlates with poor prognosis and cisplatin resistance, indicating its role in acquired drug resistance [86–88]. Li et al. reported significantly elevated *NES* expression in CRC tissues compared to normal tissues, showing that *NES* knockdown inhibits proliferation and migration of CRC cells. Their findings suggest that NES plays a critical role in promoting CRC growth and metastasis [89]. BDNF activates tropomyosin-related receptor kinase B (TrkB), initiating critical signaling pathways such as RAS/MAPK, PI3K/Akt, and JAK2/STAT3, which influence cancer progression and affect cellular processes including proliferation and migration [90, 91]. *PXDN* dysregulation is observed in various cancers, influencing the tumor microenvironment and immune cell infiltration. Overexpression of *PXDN* correlates with increased proliferation, EMT, migration, invasion, and poor clinical outcomes [92, 93]. In pancreatic cancer, high *PXDN* expression is associated with higher IC_50_ levels for drugs, indicating resistance [94]. *ITGB4*, highly expressed in CRC tissues, correlates with decreased overall survival [95]. As an integrin molecule and cell-surface receptor, ITGB4 is responsible for extracellular matrix interactions and regulates various cell signaling pathways, including proliferation, differentiation, migration, and invasion. High *ITGB4* expression is a prognostic factor in CRC and is associated with drug resistance mechanisms in breast cancer, such as resistance to tamoxifen-induced apoptosis via the PI3K/Akt pathway and anoikis resistance through RAC1 signaling [95, 96]. *CD274*, encoding PD-L1, plays a critical role in cancer by suppressing immune responses through interaction with PD-1 on T cells [97, 98]. PD-L1 influences various cancer processes such as cell growth, metastasis, and chemotherapy resistance [99]. *CD274* expression on tumor cells, contributes to immune evasion and chemotherapy resistance, including OXP-resistance in CRC, as demonstrated by Yu et al. [99]. Exploring the role of the top ten central genes in cancer progression and drug resistance not only identifies them as key candidates for predicting cancer- and resistance-related lncRNAs but also deepens our comprehension of CRC biology, providing potential targets for therapeutic intervention.

The potential upstream regulatory miRNAs targeting the top ten genes were predicted and intersected with miRNAs obtained from RNA-seq data to identify common miRNAs and construct miRNA-mRNA pairs. Several studies have demonstrated the involvement of some of these miRNAs in chemotherapy resistance across various cancers by regulating gene expression involved in key cellular processes. For example, *miR-195* exhibits dual roles as an oncogene and tumor suppressor, influencing cell proliferation, apoptosis, metastasis, invasion, and chemosensitivity by targeting specific genes in various cancers [100]. In hepatocellular carcinoma, *miR-195-3p* contributes to OXP-resistance through the *TINCR*/*miR-195-3p*/*ST6GAL1*/NF-κB signaling axis [101]. Similarly, in gastric cancer, it contributes to OXP-resistance via the *HOTAIR*/*miR-195-5p*/*ABCG2* axis, where the lncRNA *HOTAIR* acts as a ceRNA to sponge *miR-195-5p*, thereby upregulating *ABCG2* expression and promoting OXP-resistance in cancer cells [102]. The *miR-27b-3p* and *miR-15a-5p* are implicated in OXP-resistance in CRC through distinct regulatory mechanisms. *MiR-27b-3p* enhances the sensitivity of CRC cells to OXP by suppressing autophagy via inhibition of ATG10. The regulatory axis involving c-Myc/*miR-27b-3p*/ATG10 plays a pivotal role in modulating OXP-resistance [103]. Conversely, inhibition of *miR-27b-3p* by c-Myc promotes OXP-resistance, underscoring the critical role of *miR-27b-3p* in this context. On the other hand, *miR-15a-5p* contributes to OXP-resistance in CRC cells through the SIRT4 axis. This axis affects multiple signaling pathways including STAT3/TWIST1 and PETN/Akt, which are known to influence cellular responses to chemotherapy [104]. The *miR-181a-5p*/*miR-382-5p*/*CELF1* axis is pivotal in regulating cisplatin resistance in lung squamous cell carcinoma. The lncRNA *DLX6-AS1* negatively correlates with *miR-181a-5p* and *miR-382-5p* expression, thereby modulating *CELF1* expression and contributing to drug resistance [105]. Additionally, lncRNAs *CRNDE*, *ANRIL*, and *CASC15* interact with *miR-181a-5p* to promote OXP-resistance in CRC through ATP-binding protein activation [106]. *MiR-181a-5p* indirectly binds to glucose-related protein (*GRP78*), promoting tumor progression and OXP-resistance. Low *miR-181a* expression is significantly associated with cervical cancer growth and OXP-resistance [107]. *MiR-205* exhibits a dual role as either an oncogene or a tumor suppressor depending on the cancer type and specific targets [108]. It influences proliferation, tumor progression, and invasion. In some contexts, *miR-205* is targeted by lncRNA *NEAT1*, affecting the *miR-205-5p*/VEGFA axis [109], and by lncRNA *ZEB1-AS1*, influencing the *miR-205*/YAP1 axis [110]. Other miRNAs such as *miR-30a* [111], *miR-23a* [112] and *miR-23b* [113], *miR-329* [114], *miR-130* [115], *miR-192* [116] and *miR-190* [117] also impact various cellular processes by targeting specific genes, contributing to CRC progression and drug resistance.

The miRNAs target mRNAs to modulate their expression, whereas lncRNAs target miRNAs, thereby regulating both miRNAs and their downstream target genes [118]. lncRNAs directly or indirectly modulate expression of genes [3]. Mounting evidence suggests that analyzing the intricate lncRNA-miRNA-mRNA regulatory network provides insights into the molecular mechanisms underlying chemotherapy resistance, introducing lncRNAs as novel biomarkers for diagnosis and potential targets for drug-resistant cancers [119, 120]. In the present study, lncRNAs were predicted based on their interactions with miRNAs, and subsequently, an lncRNA-miRNA-mRNA network was constructed. Following this, the lncRNAs *NEAT1*, *MALAT1*, and *OIP5-AS1* were identified as key candidates potentially associated with OXP-resistance in CRC. Examining the expression patterns of the predicted lncRNAs revealed statistically significant elevations in the OXP-resistant cells compared to the parental cells. Considering that exosomes are crucial mediators in cell communication and transfer drug resistance features from resistance cells to sensitive recipient cells by carrying cargo such as lncRNAs [33, 72], we explored the expression patterns of the predicted lncRNAs in exosomes isolated from OXP-resistant cells compared to parental cells.

*NEAT1*, an oncogenic lncRNA, induces cell proliferation, metastasis, invasion, and therapy resistance, while suppresses apoptosis in cancer cells [121]. Studies have shown that a high level of *NEAT1* expression is associated with a poor OS and a poor prognosis in CRC patients [122]. *NEAT1* was reported to activate the Wnt/β-catenin signaling pathway significantly, thereby contributing to the progression of CRC [123]. This lncRNA can act as a ceRNA by sponging miRNAs, thereby targeting them to regulate mRNA and influence signaling pathways [124]. Numerous studies have reported that *NEAT1* expression is associated with chemotherapy resistance in various cancer cells and isolated exosomes. *NEAT1* has been implicated in platinum resistance in various cancers. Li et al., reported that upregulated *NEAT1* regulates OXP-resistance in gastric cancer [125]. Furthermore, Liu et al., demonstrated that *NEAT1* contributes to cisplatin resistance by modulating Rsf-1 expression and the Ras-MAPK pathway in nasopharyngeal carcinoma [126]. Zhu et al. reported that the *NEAT1*/*miR-770-5p*/PARP1 axis mediates cisplatin resistance in ovarian cancer, with *NEAT1* regulating *PARP1* [127]. *NEAT1* also has the potential to contribute to resistance against other chemotherapeutic agents. For instance, a study demonstrated that *NEAT1* promotes EMT and sorafenib resistance in renal cell carcinoma through the *miR-34a*/c-Met axis [128]. *NEAT1* was also found to promote 5-FU resistance and modulate autophagy in CRC by targeting *miR-34a* [23]. Its expression was also shown to be elevated in paclitaxel-resistant breast cancer cell lines and their isolated exosomes. The study reported that *NEAT1*, through targeting *miR-133b*, modulates *CXCL12* and promotes migration, and induces resistant to paclitaxel cells [129]. Our study demonstrated significantly elevated levels of *NEAT1* in OXP-resistant cells compared to parental cells, consistent with previous findings in other types of cancer This study may be the first to demonstrate the association of *NEAT1* with OXP-resistance in CRC cells and to identify its exosomal form in isolated exosomes, which could potentially serve as a biomarker. However, further investigation is necessary to fully comprehend the mechanism by which *NEAT1*, including its exosomal form, contributes to OXP-resistance.

*MALAT1*, known for its oncogenic properties, has emerged as a critical player in several cancers, including CRC, where it enhances cell proliferation, angiogenesis, migration, and invasion while suppressing apoptosis [130]. This lncRNA contributes to CRC tumorigenesis and progression by modulating critical signaling pathways, such as Wnt/β-catenin and PI3K/Akt, and by targeting miRNAs that impact essential cellular processes [131, 132]. For example, a study has shown that *MALAT1* contributes to the promotion of proliferation and invasion in CRC via regulating the *miR-508-5p/*RAB14 axis, which in *RAB14*, a member of the RAS oncogene family [133]. Similar to *NEAT1*, evidence has shown that *MALAT1* is frequently overexpressed in CRC tissues and implicated in the formation of nuclear speckle bodies, correlating with poor disease prognosis [134]. Notably, its upregulation is implicated in conferring resistance to platinum-based chemotherapies like OXP and cisplatin. For example, *MALAT1* promotes resistance to cisplatin in cervical cancer by inhibiting apoptosis through activation of the PI3K/Akt pathway [135]. Furthermore, it can enhance the development of cisplatin resistance in lung cancer, ultimately resulting in a significantly poor prognosis [136]. Li et al. reported that increased *MALAT1* expression correlated with decreased survival rates and poorer response to oxaliplatin-based chemotherapy in advanced CRC patients [137]. Wei et al. demonstrated that *MALAT1* promotes OXP-resistance in HCT116 cell by influencing the JNK pathway cells [138]. Previous studies have demonstrated that the intercellular transfer of *MALAT1* through exosomes enhances proliferation, invasion, and metastasis in various cancers. For instance, in a study, it was revealed that exosomal *MALAT1* promotes invasion and metastasis in CRC cells by regulating FUT4 and activating the PI3K/Akt/mTOR pathway [132]. In breast cancer tissues, elevated *MALAT1* levels are associated with disease progression, and exosomal *MALAT1* induces cell proliferation [139]. Studies have also reported that exosomal *MALAT1* contributes to chemotherapy resistance in cancer. Hu et al., showed that exosomal *MALAT1* regulates the *miR-370-3p*/STAT3 axis to promote cisplatin resistance in cervical cancer through the activation of the PI3K/Akt pathway [72]. However, the involvement of exosomal *MALAT1* in OXP-resistance in CRC has not yet been studied. In the present study, we observed elevated *MALAT1* expression in OXP-resistant CRC cells and their isolated exosomes. This finding, along with previous studies, underscores *MALAT1* as a potential biomarker for predicting treatment response and targeted therapeutic strategies to overcome OXP-resistance in CRC. Furthermore, our study reports, for the first time, the involvement of exosomal *MALAT1* in OXP-resistance in CRC.

Similarly, emerging evidence has demonstrated that dysregulation of *OIP5-AS1* contributes to cancer progression and drug resistance across various cancers [140–142]. In gastric cancer, *OIP5-AS1* modulates the *miR-367-3p*/HMGA2 axis to regulate the Wnt/β-catenin and PI3K/Akt pathways, promoting cancer progression [143]. Studies have shown that high expression level of this lncRNA correlate with poor overall survival, which indicates a poor prognosis [142]. *OIP5-AS1* regulates cell proliferation, cell cycle, colony formation, apoptosis, migration, invasion, and resistance to chemotherapy and radiotherapy by targeting miRNAs and modulating signaling pathways [144]. Liang et al. reported high expression of *OIP5-AS1* in cancer tissues and OXP-resistant cells compared to parental cells, consistent with our findings in the HCT116/OXR sub-cell line [145]. In osteosarcoma, *OIP5-AS1* contributes to cisplatin resistance by inducing the LPAATβ/PI3K/Akt/mTOR pathway [146]. Exosomal *OIP5-AS1* has been demonstrated to promote resistance to trastuzumab in breast cancer through the *miR-381-3p*/HMGB3 axis [147]. Our study also demonstrated high levels of this lncRNA in exosomes derived from OXP-resistant CRC cells. This study is the first to establish a link between exosomal *OIP5-AS1* and resistance to OXP, suggesting its potential as a biomarker.

Overall, our study has provided new insights into predicting key lncRNAs and has introduced *NEAT1*, *MALAT1*, and *OIP5-AS1* associated with OXP-resistance in CRC. The limitations of our study include the need to involve diverse CRC cell lines with different levels of OXP-resistance, as well as in-vivo experiment. Further experiments should explore the mechanisms by which these lncRNAs target miRNAs and regulate gene expression within our constructed ceRNA regulatory network. Additionally, it is essential to investigate their involvement in the signaling pathways predicted in this study. Our study showed that these lncRNAs are present at high levels in exosomes derived from OXP-resistant CRC cells, which highlights the need for future studies to understand the mechanisms of their packaging into exosomes. By exploring the roles of exosomal *NEAT1*, *MALAT1*, and *OIP5-AS1*, we could develop personalized treatment strategies.

## Conclusion

*In-silico* approaches applied to the analysis of omics data enable the study of regulatory networks and the identification of predictive biomarkers for cancer diagnosis, progression, treatment, and prevention [148, 149]. Collectively, *NEAT1*, *MALAT1*, and *OIP5-AS1* lncRNAs are crucial contributors to the development of CRC and hold promise as biomarkers for predicting OXP-resistance. In this study, for the first time, we revealed the expression patterns of *NEAT1* in both cells and exosomes, as well as *MALAT1* and *OIP5-AS1* in the exosomes of OXP-resistant HCT116 cells. To uncover the intricate molecular mechanisms of these lncRNAs, additional *in-vitro* and *in-vivo* experiments are essential.

## Supporting information

S1 Table List of DEGs and DElncRNAs identified from GSE42387. (XLSX)

S2 Table Functional enrichment analysis of oxaliplatin resistance-associated DEGs. (XLSX)

S3 Table List of predicted miRNAs from TarBase and miRTarBase databases. (XLSX)

S4 Table List of DEmiRNAs identified from GSE119481. (XLSX)

S5 Table List of miRNAs overlapped between the predicted miRNAs and the DEmiRNAs from GSE119481. (XLSX)

S6 Table List of predicted lncRNAs. (XLSX)

S7 Table Enrichment analysis of predicted lncRNAs through LncSEA. (XLSX)

S8 Table Pathway analysis of genes associated with key lncRNAs. (XLSX)

S1 Raw images (PDF)

S2 Raw images (PDF)

S3 Raw images (PDF)

## References

[1] Sung H, Ferlay J, Siegel RL, Laversanne M, Soerjomataram I, Jemal A, et al. Global Cancer Statistics 2020: GLOBOCAN Estimates of Incidence and Mortality Worldwide for 36 Cancers in 185 Countries. CA: a cancer journal for clinicians. 2021;71(3):209–49. doi: 10.3322/caac.21660 33538338

[2] Dutta A, Pratiti R, Kalantary A, Aboulian A, Shekherdimian S. Colorectal Cancer: A Systematic Review of the Current Situation and Screening in North and Central Asian Countries. Cureus. 2023;15(1):e33424. doi: 10.7759/cureus.33424 36751203 PMC9899155

[3] Ahmad R, Singh JK, Wunnava A, Al-Obeed O, Abdulla M, Srivastava SK. Emerging trends in colorectal cancer: Dysregulated signaling pathways (Review). International journal of molecular medicine. 2021;47(3). doi: 10.3892/ijmm.2021.4847 33655327 PMC7834960

[4] Dekker E, Tanis PJ, Vleugels JLA, Kasi PM, Wallace MB. Colorectal cancer. Lancet (London, England). 2019;394(10207):1467–80. doi: 10.1016/S0140-6736(19)32319-0 31631858

[5] Wang D, Lippard SJ. Cellular processing of platinum anticancer drugs. Nature reviews Drug discovery. 2005;4(4):307–20. 15789122 10.1038/nrd1691

[6] Hammond WA, Swaika A, Mody K. Pharmacologic resistance in colorectal cancer: a review. Therapeutic advances in medical oncology. 2016;8(1):57–84. doi: 10.1177/1758834015614530 26753006 PMC4699262

[7] Mauri G, Gori V, Bonazzina E, Amatu A, Tosi F, Bencardino K, et al. Oxaliplatin retreatment in metastatic colorectal cancer: Systematic review and future research opportunities. Cancer Treatment Reviews. 2020;91:102112. doi: 10.1016/j.ctrv.2020.102112 33091698

[8] Huang WS, Hsieh MC, Huang CY, Kuo YH, Tung SY, Shen CH, et al. The Association of CXC Receptor 4 Mediated Signaling Pathway with Oxaliplatin-Resistant Human Colorectal Cancer Cells. PloS one. 2016;11(9):e0159927. doi: 10.1371/journal.pone.0159927 27668882 PMC5036794

[9] William-Faltaos S, Rouillard D, Lechat P, Bastian G. Cell cycle arrest by oxaliplatin on cancer cells. Fundamental & clinical pharmacology. 2007;21(2):165–72. doi: 10.1111/j.1472-8206.2007.00462.x 17391289

[10] Noordhuis P, Laan AC, van de Born K, Losekoot N, Kathmann I, Peters GJ. Oxaliplatin activity in selected and unselected human ovarian and colorectal cancer cell lines. Biochemical pharmacology. 2008;76(1):53–61. doi: 10.1016/j.bcp.2008.04.007 18508032

[11] Cheraghi-Shavi T, Jalal R, Minuchehr Z. TGM2, HMGA2, FXYD3, and LGALS4 genes as biomarkers in acquired oxaliplatin resistance of human colorectal cancer: A systems biology approach. PloS one. 2023;18(8):e0289535. doi: 10.1371/journal.pone.0289535 37535601 PMC10399784

[12] Lin Q, Luo L, Wang H. A New Oxaliplatin Resistance-Related Gene Signature With Strong Predicting Ability in Colon Cancer Identified by Comprehensive Profiling. Frontiers in oncology. 2021;11:644956. doi: 10.3389/fonc.2021.644956 34026619 PMC8138443

[13] O’Dowd PD, Sutcliffe DF, Griffith DM. Oxaliplatin and its derivatives–An overview. Coordination Chemistry Reviews. 2023;497:215439.

[14] Luo ZD, Wang YF, Zhao YX, Yu LC, Li T, Fan YJ, et al. Emerging roles of non-coding RNAs in colorectal cancer oxaliplatin resistance and liquid biopsy potential. World J Gastroenterol. 2023;29(1):1–18. doi: 10.3748/wjg.v29.i1.1 36683709 PMC9850945

[15] Statello L, Guo C-J, Chen L-L, Huarte M. Gene regulation by long non-coding RNAs and its biological functions. Nature Reviews Molecular Cell Biology. 2021;22(2):96–118. doi: 10.1038/s41580-020-00315-9 33353982 PMC7754182

[16] Sun B, Liu C, Li H, Zhang L, Luo G, Liang S, et al. Research progress on the interactions between long non-coding RNAs and microRNAs in human cancer. Oncology letters. 2020;19(1):595–605. doi: 10.3892/ol.2019.11182 31897175 PMC6923957

[17] Siddiqui H, Al-Ghafari A, Choudhry H, Al Doghaither H. Roles of long non-coding RNAs in colorectal cancer tumorigenesis: A Review. Molecular and clinical oncology. 2019;11(2):167–72. doi: 10.3892/mco.2019.1872 31281651 PMC6589935

[18] Yang Y, Junjie P, Sanjun C, Ma Y. Long non-coding RNAs in colorectal cancer: progression and future directions. Journal of Cancer. 2017;8(16):3212. doi: 10.7150/jca.19794 29158793 PMC5665037

[19] He Q, Long J, Yin Y, Li Y, Lei X, Li Z, et al. Emerging roles of lncRNAs in the formation and progression of colorectal cancer. Frontiers in oncology. 2020;9:1542. doi: 10.3389/fonc.2019.01542 32010629 PMC6978842

[20] Zhang W, Guan X, Tang J. The long non-coding RNA landscape in triple-negative breast cancer. Cell proliferation. 2021;54(2):e12966. doi: 10.1111/cpr.12966 33314471 PMC7848969

[21] Fan C, Yuan Q, Liu G, Zhang Y, Yan M, Sun Q, et al. Long non-coding RNA MALAT1 regulates oxaliplatin-resistance via miR-324-3p/ADAM17 axis in colorectal cancer cells. Cancer cell international. 2020;20:473. doi: 10.1186/s12935-020-01549-5 33005106 PMC7525982

[22] Azizidoost S, Ghaedrahmati F, Anbiyaee O, Ahmad Ali R, Cheraghzadeh M, Farzaneh M. Emerging roles for lncRNA-NEAT1 in colorectal cancer. Cancer cell international. 2022;22(1):209. doi: 10.1186/s12935-022-02627-6 35676702 PMC9178824

[23] Liu F, Ai FY, Zhang DC, Tian L, Yang ZY, Liu SJ. LncRNA NEAT1 knockdown attenuates autophagy to elevate 5-FU sensitivity in colorectal cancer via targeting miR-34a. Cancer medicine. 2020;9(3):1079–91. doi: 10.1002/cam4.2746 31802650 PMC6997058

[24] Weng X, Liu H, Ruan J, Du M, Wang L, Mao J, et al. HOTAIR/miR-1277-5p/ZEB1 axis mediates hypoxia-induced oxaliplatin resistance via regulating epithelial-mesenchymal transition in colorectal cancer. Cell Death Discovery. 2022;8(1):310. doi: 10.1038/s41420-022-01096-0 35798695 PMC9263107

[25] Yue B, Cai D, Liu C, Fang C, Yan D. Linc00152 functions as a competing endogenous RNA to confer oxaliplatin resistance and holds prognostic values in colon cancer. Molecular therapy. 2016;24(12):2064–77. doi: 10.1038/mt.2016.180 27633443 PMC5167786

[26] Gao H, Song X, Kang T, Yan B, Feng L, Gao L, et al. Long noncoding RNA CRNDE functions as a competing endogenous RNA to promote metastasis and oxaliplatin resistance by sponging miR-136 in colorectal cancer. OncoTargets and therapy. 2017;10:205–16. doi: 10.2147/OTT.S116178 28115855 PMC5221653

[27] Meng X, Sun W, Yu J, Zhou Y, Gu Y, Han J, et al. LINC00460-miR-149-5p/miR-150-5p-Mutant p53 Feedback Loop Promotes Oxaliplatin Resistance in Colorectal Cancer. Molecular therapy Nucleic acids. 2020;22:1004–15. doi: 10.1016/j.omtn.2020.10.018 33251049 PMC7679243

[28] Li Y, Li C, Li D, Yang L, Jin J, Zhang B. lncRNA KCNQ1OT1 enhances the chemoresistance of oxaliplatin in colon cancer by targeting the miR-34a/ATG4B pathway. OncoTargets and therapy. 2019;12:2649–60. doi: 10.2147/OTT.S188054 31040703 PMC6462170

[29] Sun J, Zhou H, Bao X, Wu Y, Jia H, Zhao H, et al. lncRNA TUG1 Facilitates Colorectal Cancer Stem Cell Characteristics and Chemoresistance by Enhancing GATA6 Protein Stability. Stem cells international. 2021;2021:1075481. doi: 10.1155/2021/1075481 34858502 PMC8632465

[30] Li L, Shang J, Zhang Y, Liu S, Peng Y, Zhou Z, et al. MEG3 is a prognostic factor for CRC and promotes chemosensitivity by enhancing oxaliplatin-induced cell apoptosis. Oncology reports. 2017;38(3):1383–92. doi: 10.3892/or.2017.5828 28731151 PMC5549059

[31] Wu S, Yang X, Tang W, Familiari G, Relucenti M, Aschner M, et al. Chemotherapeutic Risk lncRNA-PVT1 SNP Sensitizes Metastatic Colorectal Cancer to FOLFOX Regimen. Frontiers in oncology. 2022;12:808889. doi: 10.3389/fonc.2022.808889 35433465 PMC9008320

[32] Li Y, Gan Y, Liu J, Li J, Zhou Z, Tian R, et al. Downregulation of MEIS1 mediated by ELFN1-AS1/EZH2/DNMT3a axis promotes tumorigenesis and oxaliplatin resistance in colorectal cancer. Signal transduction and targeted therapy. 2022;7(1):87. doi: 10.1038/s41392-022-00902-6 35351858 PMC8964798

[33] Li S, Yi M, Dong B, Jiao Y, Luo S, Wu K. The roles of exosomes in cancer drug resistance and its therapeutic application. Clinical and translational medicine. 2020;10(8):e257. doi: 10.1002/ctm2.257 33377643 PMC7752167

[34] Mahmoudi-Aznaveh A, Tavoosidana G, Najmabadi H, Azizi Z, Ardestani A. The liver-derived exosomes stimulate insulin gene expression in pancreatic beta cells under condition of insulin resistance. Frontiers in Endocrinology. 2023;14. doi: 10.3389/fendo.2023.1303930 38027137 PMC10661932

[35] Wei H, Chen Q, Lin L, Sha C, Li T, Liu Y, et al. Regulation of exosome production and cargo sorting. International journal of biological sciences. 2021;17(1):163–77. doi: 10.7150/ijbs.53671 33390841 PMC7757038

[36] Krylova SV, Feng D. The machinery of exosomes: biogenesis, release, and uptake. International journal of molecular sciences. 2023;24(2):1337. doi: 10.3390/ijms24021337 36674857 PMC9865891

[37] Ren B, Li X, Zhang Z, Tai S, Yu S. Exosomes: a significant medium for regulating drug resistance through cargo delivery. Frontiers in Molecular Biosciences. 2024;11. doi: 10.3389/fmolb.2024.1379822 39135913 PMC11317298

[38] Li J, Gao N, Gao Z, Liu W, Pang B, Dong X, et al. The Emerging Role of Exosomes in Cancer Chemoresistance. Frontiers in Cell and Developmental Biology. 2021;9. doi: 10.3389/fcell.2021.737962 34778252 PMC8581179

[39] Ye Y, Li Y, Wu C, Shan Y, Li J, Jiang D, et al. Exosomes Mediate the Production of Oxaliplatin Resistance and Affect Biological Behaviors of Colon Cancer Cell Lines. Current cancer drug targets. 2024. doi: 10.2174/0115680096298783240517050259 38956907

[40] Stefańska K, Józkowiak M, Angelova Volponi A, Shibli JA, Golkar-Narenji A, Antosik P, et al. The Role of Exosomes in Human Carcinogenesis and Cancer Therapy-Recent Findings from Molecular and Clinical Research. Cells. 2023;12(3). doi: 10.3390/cells12030356 36766698 PMC9913699

[41] Ren J, Ding L, Zhang D, Shi G, Xu Q, Shen S, et al. Carcinoma-associated fibroblasts promote the stemness and chemoresistance of colorectal cancer by transferring exosomal lncRNA H19. Theranostics. 2018;8(14):3932–48. doi: 10.7150/thno.25541 30083271 PMC6071523

[42] Deng X, Ruan H, Zhang X, Xu X, Zhu Y, Peng H, et al. Long noncoding RNA CCAL transferred from fibroblasts by exosomes promotes chemoresistance of colorectal cancer cells. International journal of cancer. 2020;146(6):1700–16. doi: 10.1002/ijc.32608 31381140

[43] Lin S, Zhu B. Exosome-transmitted FOSL1 from cancer-associated fibroblasts drives colorectal cancer stemness and chemo-resistance through transcriptionally activating ITGB4. Molecular and Cellular Biochemistry. 2024;479(3):665–77. doi: 10.1007/s11010-023-04737-9 37160555

[44] Sun R, He XY, Mei C, Ou CL. Role of exosomal long non-coding RNAs in colorectal cancer. World journal of gastrointestinal oncology. 2021;13(8):867–78. doi: 10.4251/wjgo.v13.i8.867 34457192 PMC8371516

[45] Zhu S, Mao J, Zhang X, Wang P, Zhou Y, Tong J, et al. CAF-derived exosomal lncRNA FAL1 promotes chemoresistance to oxaliplatin by regulating autophagy in colorectal cancer. Digestive and liver disease: official journal of the Italian Society of Gastroenterology and the Italian Association for the Study of the Liver. 2024;56(2):330–42. 37400281 10.1016/j.dld.2023.06.010

[46] Chen X, Liu Y, Zhang Q, Liu B, Cheng Y, Zhang Y, et al. Exosomal Long Non-coding RNA HOTTIP Increases Resistance of Colorectal Cancer Cells to Mitomycin via Impairing MiR-214-Mediated Degradation of KPNA3. Frontiers in cell and developmental biology. 2020;8:582723. doi: 10.3389/fcell.2020.582723 33585440 PMC7876302

[47] Jensen NF, Stenvang J, Beck MK, Hanáková B, Belling KC, Do KN, et al. Establishment and characterization of models of chemotherapy resistance in colorectal cancer: Towards a predictive signature of chemoresistance. Molecular oncology. 2015;9(6):1169–85. doi: 10.1016/j.molonc.2015.02.008 25759163 PMC5528766

[48] Gasiulė S, Dreize N, Kaupinis A, Ražanskas R, Čiupas L, Stankevičius V, et al. Molecular Insights into miRNA-Driven Resistance to 5-Fluorouracil and Oxaliplatin Chemotherapy: miR-23b Modulates the Epithelial–Mesenchymal Transition of Colorectal Cancer Cells. Journal of clinical medicine. 2019;8(12):2115. 31810268 10.3390/jcm8122115 PMC6947029

[49] Love MI, Huber W, Anders S. Moderated estimation of fold change and dispersion for RNA-seq data with DESeq2. Genome Biology. 2014;15(12):550. doi: 10.1186/s13059-014-0550-8 25516281 PMC4302049

[50] Szklarczyk D, Kirsch R, Koutrouli M, Nastou K, Mehryary F, Hachilif R, et al. The STRING database in 2023: protein-protein association networks and functional enrichment analyses for any sequenced genome of interest. Nucleic Acids Res. 2023;51(D1):D638–d46. doi: 10.1093/nar/gkac1000 36370105 PMC9825434

[51] Shannon P, Markiel A, Ozier O, Baliga NS, Wang JT, Ramage D, et al. Cytoscape: a software environment for integrated models of biomolecular interaction networks. Genome Res. 2003;13(11):2498–504. doi: 10.1101/gr.1239303 14597658 PMC403769

[52] Sherman BT, Hao M, Qiu J, Jiao X, Baseler MW, Lane HC, et al. DAVID: a web server for functional enrichment analysis and functional annotation of gene lists (2021 update). Nucleic Acids Res. 2022;50(W1):W216–w21. 35325185 10.1093/nar/gkac194 PMC9252805

[53] Huang da W, Sherman BT, Lempicki RA. Systematic and integrative analysis of large gene lists using DAVID bioinformatics resources. Nature protocols. 2009;4(1):44–57. doi: 10.1038/nprot.2008.211 19131956

[54] Tang Y, Li M, Wang J, Pan Y, Wu FX. CytoNCA: a cytoscape plugin for centrality analysis and evaluation of protein interaction networks. Biosystems. 2015;127:67–72. doi: 10.1016/j.biosystems.2014.11.005 25451770

[55] Huang HY, Lin YC, Cui S, Huang Y, Tang Y, Xu J, et al. miRTarBase update 2022: an informative resource for experimentally validated miRNA-target interactions. Nucleic Acids Res. 2022;50(D1):D222–d30. doi: 10.1093/nar/gkab1079 34850920 PMC8728135

[56] Karagkouni D, Paraskevopoulou MD, Chatzopoulos S, Vlachos IS, Tastsoglou S, Kanellos I, et al. DIANA-TarBase v8: a decade-long collection of experimentally supported miRNA-gene interactions. Nucleic Acids Res. 2018;46(D1):D239–d45. doi: 10.1093/nar/gkx1141 29156006 PMC5753203

[57] Paraskevopoulou MD, Karagkouni D, Vlachos IS, Tastsoglou S, Hatzigeorgiou AG. microCLIP super learning framework uncovers functional transcriptome-wide miRNA interactions. Nature Communications. 2018;9(1):3601. doi: 10.1038/s41467-018-06046-y 30190538 PMC6127135

[58] Karagkouni D, Paraskevopoulou MD, Tastsoglou S, Skoufos G, Karavangeli A, Pierros V, et al. DIANA-LncBase v3: indexing experimentally supported miRNA targets on non-coding transcripts. Nucleic Acids Res. 2020;48(D1):D101–d10. doi: 10.1093/nar/gkz1036 31732741 PMC7145509

[59] Li JH, Liu S, Zhou H, Qu LH, Yang JH. starBase v2.0: decoding miRNA-ceRNA, miRNA-ncRNA and protein-RNA interaction networks from large-scale CLIP-Seq data. Nucleic acids research. 2014;42(Database issue):D92–7. doi: 10.1093/nar/gkt1248 24297251 PMC3964941

[60] Chen J, Zhang J, Gao Y, Li Y, Feng C, Song C, et al. LncSEA: a platform for long non-coding RNA related sets and enrichment analysis. Nucleic Acids Res. 2021;49(D1):D969–d80. doi: 10.1093/nar/gkaa806 33045741 PMC7778898

[61] Győrffy B. Discovery and ranking of the most robust prognostic biomarkers in serous ovarian cancer. Geroscience. 2023;45(3):1889–98. doi: 10.1007/s11357-023-00742-4 36856946 PMC10400493

[62] Kovács SA, Fekete JT, Győrffy B. Predictive biomarkers of immunotherapy response with pharmacological applications in solid tumors. Acta Pharmacol Sin. 2023;44(9):1879–89. doi: 10.1038/s41401-023-01079-6 37055532 PMC10462766

[63] McDermott M, Eustace A, Busschots S, Breen L, Clynes M, O’Donovan N, et al. In vitro Development of Chemotherapy and Targeted Therapy Drug-Resistant Cancer Cell Lines: A Practical Guide with Case Studies. Frontiers in oncology. 2014;4. 10.3389/fonc.2014.00040 PMC3944788 24639951

[64] Eke I, Cordes N. Focal adhesion signaling and therapy resistance in cancer. Seminars in cancer biology. 2015;31:65–75. doi: 10.1016/j.semcancer.2014.07.009 25117005

[65] Zhang Y, Liu X, Zhang J, Xu Y, Shao J, Hu Y, et al. Inhibition of miR-19a partially reversed the resistance of colorectal cancer to oxaliplatin via PTEN/PI3K/AKT pathway. Aging. 2020;12(7):5640–50. doi: 10.18632/aging.102929 32209726 PMC7185119

[66] Chen Y, Deng G, Fu Y, Han Y, Guo C, Yin L, et al. FOXC2 Promotes Oxaliplatin Resistance by Inducing Epithelial-Mesenchymal Transition via MAPK/ERK Signaling in Colorectal Cancer. OncoTargets and therapy. 2020;13:1625–35. doi: 10.2147/OTT.S241367 32110058 PMC7041600

[67] Nersisyan S, Novosad V, Engibaryan N, Ushkaryov Y, Nikulin S, Tonevitsky A. ECM-Receptor Regulatory Network and Its Prognostic Role in Colorectal Cancer. Front Genet. 2021;12:782699. doi: 10.3389/fgene.2021.782699 34938324 PMC8685507

[68] Janiszewska M, Primi MC, Izard T. Cell adhesion in cancer: Beyond the migration of single cells. Journal of Biological Chemistry. 2020;295(8):2495–505. doi: 10.1074/jbc.REV119.007759 31937589 PMC7039572

[69] Fu D, Hu Z, Xu X, Dai X, Liu Z. Key signal transduction pathways and crosstalk in cancer: Biological and therapeutic opportunities. Translational oncology. 2022;26:101510. doi: 10.1016/j.tranon.2022.101510 36122506 PMC9486121

[70] Gutschner T, Diederichs S. The hallmarks of cancer: a long non-coding RNA point of view. RNA Biol. 2012;9(6):703–19. doi: 10.4161/rna.20481 22664915 PMC3495743

[71] Leandro G, Erika Z, Carolina M, Jéssica B, Daniela G, Jaqueline O. lncRNAs in Hallmarks of Cancer and Clinical Applications. In: Lütfi T, Sümer A, Esen T, editors. Non-Coding RNAs. Rijeka: IntechOpen; 2019. p. Ch. 8.

[72] Hu Y, Li G, Ma Y, Luo G, Wang Q, Zhang S. Effect of Exosomal lncRNA MALAT1/miR-370-3p/STAT3 Positive Feedback Loop on PI3K/Akt Pathway Mediating Cisplatin Resistance in Cervical Cancer Cells. Journal of oncology. 2023;2023:6341011. doi: 10.1155/2023/6341011 36793374 PMC9925267

[73] Pang X, Xie R, Zhang Z, Liu Q, Wu S, Cui Y. Identification of SPP1 as an Extracellular Matrix Signature for Metastatic Castration-Resistant Prostate Cancer. Frontiers in oncology. 2019;9:924. doi: 10.3389/fonc.2019.00924 31620371 PMC6760472

[74] Gao W, Liu D, Sun H, Shao Z, Shi P, Li T, et al. SPP1 is a prognostic related biomarker and correlated with tumor-infiltrating immune cells in ovarian cancer. BMC cancer. 2022;22(1):1367. 36585688 10.1186/s12885-022-10485-8 PMC9805166

[75] Asare-Werehene M, Nakka K, Reunov A, Chiu C-T, Lee W-T, Abedini MR, et al. The exosome-mediated autocrine and paracrine actions of plasma gelsolin in ovarian cancer chemoresistance. Oncogene. 2020;39(7):1600–16. doi: 10.1038/s41388-019-1087-9 31700155 PMC7018662

[76] Wei T, Bi G, Bian Y, Ruan S, Yuan G, Xie H, et al. The Significance of Secreted Phosphoprotein 1 in Multiple Human Cancers. Frontiers in molecular biosciences. 2020;7:565383. doi: 10.3389/fmolb.2020.565383 33324676 PMC7724571

[77] Yi J, Liu Y, Zhang L, Fang C. Secreted phosphoprotein-1 accelerates the progression of human colorectal cancer through activating β-catenin signaling. Oncology letters. 2021;21(5):372. 33777196 10.3892/ol.2021.12633 PMC7988691

[78] Zhao K, Ma Z, Zhang W. Comprehensive Analysis to Identify SPP1 as a Prognostic Biomarker in Cervical Cancer. Front Genet. 2021;12:732822. doi: 10.3389/fgene.2021.732822 35058964 PMC8764398

[79] Tang H, Chen J, Han X, Feng Y, Wang F. Upregulation of SPP1 Is a Marker for Poor Lung Cancer Prognosis and Contributes to Cancer Progression and Cisplatin Resistance. Frontiers in cell and developmental biology. 2021;9:646390. doi: 10.3389/fcell.2021.646390 33996808 PMC8116663

[80] Chen X, Xiong D, Ye L, Yang H, Mei S, Wu J, et al. SPP1 inhibition improves the cisplatin chemo-sensitivity of cervical cancer cell lines. Cancer chemotherapy and pharmacology. 2019;83(4):603–13. doi: 10.1007/s00280-018-3759-5 30627777

[81] Zhang X, Zhang L, Tan X, Lin Y, Han X, Wang H, et al. Systematic analysis of genes involved in oral cancer metastasis to lymph nodes. Cellular & molecular biology letters. 2018;23:53. doi: 10.1186/s11658-018-0120-2 30459815 PMC6237046

[82] Liang W, Liu D, Wu J. c-JUN-induced upregulation of LINC00174 contributes to colorectal cancer proliferation and invasion through accelerating USP21 expression. Cell biology international. 2023;47(11):1782–98. doi: 10.1002/cbin.12069 37434557

[83] Zhang Z, Zhang Y, Qin X, Wang Y, Fu J. FGF9 promotes cisplatin resistance in colorectal cancer via regulation of Wnt/β-catenin signaling pathway. Experimental and therapeutic medicine. 2020;19(3):1711–8. 32104224 10.3892/etm.2019.8399 PMC7026987

[84] Chen T-M, Shih Y-H, Tseng JT, Lai M-C, Wu C-H, Li Y-H, et al. Overexpression of FGF9 in colon cancer cells is mediated by hypoxia-induced translational activation. Nucleic Acids Research. 2013;42(5):2932–44. doi: 10.1093/nar/gkt1286 24334956 PMC3950685

[85] Makondi PT, Chu C-M, Wei P-L, Chang Y-J. Prediction of novel target genes and pathways involved in irinotecan-resistant colorectal cancer. PloS one. 2017;12(7):e0180616. doi: 10.1371/journal.pone.0180616 28749961 PMC5531462

[86] Szymańska-Chabowska A, Świątkowski F, Jankowska-Polańska B, Mazur G, Chabowski M. Nestin Expression as a Diagnostic and Prognostic Marker in Colorectal Cancer and Other Tumors. Clinical Medicine Insights Oncology. 2021;15:11795549211038256. doi: 10.1177/11795549211038256 34421318 PMC8377314

[87] Jia R, Song L, Fei Z, Qin C, Zhao Q. Long noncoding RNA Ftx regulates the protein expression profile in HCT116 human colon cancer cells. Proteome science. 2022;20(1):7. doi: 10.1186/s12953-022-00187-1 35490216 PMC9055732

[88] Sone K, Maeno K, Masaki A, Kunii E, Takakuwa O, Kagawa Y, et al. Nestin Expression Affects Resistance to Chemotherapy and Clinical Outcome in Small Cell Lung Cancer. Frontiers in oncology. 2020;10:1367. doi: 10.3389/fonc.2020.01367 32903755 PMC7438916

[89] Li J, Wang R, Yang L, Wu Q, Wang Q, Nie Z, et al. Knockdown of Nestin inhibits proliferation and migration of colorectal cancer cells. International journal of clinical and experimental pathology. 2015;8(6):6377. 26261513 PMC4525847

[90] Tanaka K, Okugawa Y, Toiyama Y, Inoue Y, Saigusa S, Kawamura M, et al. Brain-derived neurotrophic factor (BDNF)-induced tropomyosin-related kinase B (Trk B) signaling is a potential therapeutic target for peritoneal carcinomatosis arising from colorectal cancer. PloS one. 2014;9(5):e96410. doi: 10.1371/journal.pone.0096410 24801982 PMC4011754

[91] Serafim V Junior, Fernandes GMdM, Oliveira-Cucolo JGd, Pavarino EC, Goloni-Bertollo EM. Role of Tropomyosin-related kinase B receptor and brain-derived neurotrophic factor in cancer. Cytokine. 2020;136:155270. doi: 10.1016/j.cyto.2020.155270 32911446

[92] Wyllie K, Panagopoulos V, Cox TR. The role of peroxidasin in solid cancer progression. Biochemical Society transactions. 2023;51(5):1881–95. doi: 10.1042/BST20230018 37801286 PMC10657184

[93] Zheng YZ, Liang L. High expression of PXDN is associated with poor prognosis and promotes proliferation, invasion as well as migration in ovarian cancer. Ann Diagn Pathol. 2018;34:161–5. doi: 10.1016/j.anndiagpath.2018.03.002 29661721

[94] Zhou X, Sun Q, Xu C, Zhou Z, Chen X, Zhu X, et al. A systematic pan-cancer analysis of PXDN as a potential target for clinical diagnosis and treatment. Frontiers in oncology. 2022;12. doi: 10.3389/fonc.2022.952849 35982948 PMC9380648

[95] Jiang X, Wang J, Wang M, Xuan M, Han S, Li C, et al. ITGB4 as a novel serum diagnosis biomarker and potential therapeutic target for colorectal cancer. Cancer medicine. 2021;10(19):6823–34. doi: 10.1002/cam4.4216 34414684 PMC8495272

[96] Fang H, Ren W, Cui Q, Liang H, Yang C, Liu W, et al. Integrin β4 promotes DNA damage-related drug resistance in triple-negative breast cancer via TNFAIP2/IQGAP1/RAC1. eLife. 2023;12. 10.7554/eLife.88483 PMC10547475 37787041

[97] Masugi Y, Nishihara R, Yang J, Mima K, da Silva A, Shi Y, et al. Tumour CD274 (PD-L1) expression and T cells in colorectal cancer. Gut. 2017;66(8):1463–73. doi: 10.1136/gutjnl-2016-311421 27196573 PMC5097696

[98] Sun C, Mezzadra R, Schumacher TN. Regulation and Function of the PD-L1 Checkpoint. Immunity. 2018;48(3):434–52. doi: 10.1016/j.immuni.2018.03.014 29562194 PMC7116507

[99] Yu M, Wang H, Zhao W, Ge X, Huang W, Lin F, et al. Targeting type Iγ phosphatidylinositol phosphate kinase overcomes oxaliplatin resistance in colorectal cancer. Theranostics. 2022;12(9):4386–98. doi: 10.7150/thno.69863 35673560 PMC9169372

[100] Yu W, Liang X, Li X, Zhang Y, Sun Z, Liu Y, et al. MicroRNA-195: a review of its role in cancers. OncoTargets and therapy. 2018;11:7109–23. doi: 10.2147/OTT.S183600 30410367 PMC6200091

[101] Mei J, Lin W, Li S, Tang Y, Ye Z, Lu L, et al. Long noncoding RNA TINCR facilitates hepatocellular carcinoma progression and dampens chemosensitivity to oxaliplatin by regulating the miR-195-3p/ST6GAL1/NF-κB pathway. Journal of experimental & clinical cancer research: CR. 2022;41(1):5. 34980201 10.1186/s13046-021-02197-x PMC8722212

[102] Luo Y, Lu X, Ma W, Xiao Y, Wei C, Yuan X, et al. Dampening HOTAIR sensitizes the gastric cancer cells to oxaliplatin through miR-195-5p and ABCG2 pathway. Journal of cellular and molecular medicine. 2023;27(22):3591–600. doi: 10.1111/jcmm.17925 37621132 PMC10660622

[103] Sun W, Li J, Zhou L, Han J, Liu R, Zhang H, et al. The c-Myc/miR-27b-3p/ATG10 regulatory axis regulates chemoresistance in colorectal cancer. Theranostics. 2020;10(5):1981–96. doi: 10.7150/thno.37621 32104496 PMC7019154

[104] Deng J, Wang H, Liang Y, Zhao L, Li Y, Yan Y, et al. miR-15a-5p enhances the malignant phenotypes of colorectal cancer cells through the STAT3/TWIST1 and PTEN/AKT signaling pathways by targeting SIRT4. Cellular signalling. 2023;101:110517. doi: 10.1016/j.cellsig.2022.110517 36332797

[105] Zhao X, Wang J, Zhu R, Zhang J, Zhang Y. DLX6-AS1 activated by H3K4me1 enhanced secondary cisplatin resistance of lung squamous cell carcinoma through modulating miR-181a-5p/miR-382-5p/CELF1 axis. Scientific Reports. 2021;11(1):21014. doi: 10.1038/s41598-021-99555-8 34697393 PMC8546124

[106] Qi F-f, Yang Y, Zhang H, Chen H. Long non-coding RNAs: Key regulators in oxaliplatin resistance of colorectal cancer. Biomedicine & Pharmacotherapy. 2020;128:110329. doi: 10.1016/j.biopha.2020.110329 32502843

[107] Luo C, Qiu J. miR-181a Inhibits Cervical Cancer Development via Downregulating GRP78. Oncology research. 2017;25(8):1341–8. doi: 10.3727/096504017X14867268787969 28245171 PMC7841036

[108] Qin AY, Zhang XW, Liu L, Yu JP, Li H, Wang SZ, et al. MiR-205 in cancer: an angel or a devil? European journal of cell biology. 2013;92(2):54–60. doi: 10.1016/j.ejcb.2012.11.002 23279926

[109] Liu H, Li A, Sun Z, Zhang J, Xu H. Long non-coding RNA NEAT1 promotes colorectal cancer progression by regulating miR-205-5p/VEGFA axis. Human cell. 2020;33(2):386–96. doi: 10.1007/s13577-019-00301-0 32065361

[110] Jin Z, Chen B. LncRNA ZEB1-AS1 Regulates Colorectal Cancer Cells by MiR-205/YAP1 Axis. Open medicine (Warsaw, Poland). 2020;15:175–84. doi: 10.1515/med-2020-0026 32190742 PMC7065425

[111] Wang R, Wang J, Chen Y, Chen Y, Xi Q, Sun L, et al. Circular RNA circLDLR facilitates cancer progression by altering the miR-30a-3p/SOAT1 axis in colorectal cancer. Cell death discovery. 2022;8(1):314. doi: 10.1038/s41420-022-01110-5 35821230 PMC9276972

[112] Deng YH, Deng ZH, Hao H, Wu XL, Gao H, Tang SH, et al. MicroRNA-23a promotes colorectal cancer cell survival by targeting PDK4. Experimental cell research. 2018;373(1):171–9. doi: 10.1016/j.yexcr.2018.10.010 30342991

[113] Kou CH, Zhou T, Han XL, Zhuang HJ, Qian HX. Downregulation of mir-23b in plasma is associated with poor prognosis in patients with colorectal cancer. Oncology letters. 2016;12(6):4838–44. doi: 10.3892/ol.2016.5265 28101227 PMC5228308

[114] Ye J, Lei J, Fang Q, Shen Y, Xia W, Hu X, et al. miR-4666-3p and miR-329 synergistically suppress the stemness of colorectal cancer cells via targeting TGF-β/Smad pathway. Frontiers in oncology. 2019;9:1251. 31824844 10.3389/fonc.2019.01251 PMC6880832

[115] Shi L, Zhao Y, Liu X, Qian J, Yang X, Li W. Circular RNA circWHSC1 facilitates colorectal cancer cell proliferation by targeting miR-130a-5p/zeb1 signaling in vitro and in vivo. Heliyon. 2023;9(10):e20176. doi: 10.1016/j.heliyon.2023.e20176 37810854 PMC10556587

[116] Chen X, Su X, Lin M, Fu B, Zhou C, Ling C, et al. Expression of miR-192-5p in colon cancer serum and its relationship with clinicopathologic features. American journal of translational research. 2021;13(8):9371–6. 34540055 PMC8430106

[117] Hu X, Wang Y, Liang H, Fan Q, Zhu R, Cui J, et al. miR-23a/b promote tumor growth and suppress apoptosis by targeting PDCD4 in gastric cancer. Cell Death Dis. 2017;8(10):e3059. doi: 10.1038/cddis.2017.447 28981115 PMC5680570

[118] Lampropoulou DI, Pliakou E, Aravantinos G, Filippou D, Gazouli M. The Role of Exosomal Non-Coding RNAs in Colorectal Cancer Drug Resistance. International journal of molecular sciences. 2022;23(3). doi: 10.3390/ijms23031473 35163397 PMC8835818

[119] Kong X, Hu S, Yuan Y, Du Y, Zhu Z, Song Z, et al. Analysis of lncRNA, miRNA and mRNA-associated ceRNA networks and identification of potential drug targets for drug-resistant non-small cell lung cancer. Journal of Cancer. 2020;11(11):3357–68. doi: 10.7150/jca.40729 32231742 PMC7097957

[120] Raziq K, Cai M, Dong K, Wang P, Afrifa J, Fu S. Competitive endogenous network of lncRNA, miRNA, and mRNA in the chemoresistance of gastrointestinal tract adenocarcinomas. Biomedicine & Pharmacotherapy. 2020;130:110570. 32763816 10.1016/j.biopha.2020.110570

[121] Li K, Yao T, Zhang Y, Li W, Wang Z. NEAT1 as a competing endogenous RNA in tumorigenesis of various cancers: Role, mechanism and therapeutic potential. International journal of biological sciences. 2021;17(13):3428–40. doi: 10.7150/ijbs.62728 34512157 PMC8416723

[122] Yang C, Li Z, Li Y, Xu R, Wang Y, Tian Y, et al. Long non-coding RNA NEAT1 overexpression is associated with poor prognosis in cancer patients: a systematic review and meta-analysis. Oncotarget. 2017;8(2):2672–80. doi: 10.18632/oncotarget.13737 27926523 PMC5356832

[123] Zhang M, Weng W, Zhang Q, Wu Y, Ni S, Tan C, et al. The lncRNA NEAT1 activates Wnt/β-catenin signaling and promotes colorectal cancer progression via interacting with DDX5. J Hematol Oncol. 2018;11(1):113. 30185232 10.1186/s13045-018-0656-7 PMC6125951

[124] Zhu Y, Wang X, Zheng L, Li D, Liu Z, Teng L. The lncRNA NEAT1 Inhibits miRNA-216b and Promotes Colorectal Cancer Progression by Indirectly Activating YY1. Journal of oncology. 2022;2022:8130132. doi: 10.1155/2022/8130132 36262350 PMC9576420

[125] Li Y, Peng C, Fang C, Huang K. Upregulation of nuclear-enriched abundant transcript 1 confers oxaliplatin resistance to gastric cancer. Cell biology international. 2020;44(2):446–55. doi: 10.1002/cbin.11245 31617275

[126] Liu F, Tai Y, Ma J. LncRNA NEAT1/let-7a-5p axis regulates the cisplatin resistance in nasopharyngeal carcinoma by targeting Rsf-1 and modulating the Ras-MAPK pathway. Cancer biology & therapy. 2018;19(6):534–42. doi: 10.1080/15384047.2018.1450119 29565706 PMC5927658

[127] Zhu M, Yang L, Wang X. NEAT1 Knockdown Suppresses the Cisplatin Resistance in Ovarian Cancer by Regulating miR-770-5p/PARP1 Axis. Cancer management and research. 2020;12:7277–89. doi: 10.2147/CMAR.S257311 32884343 PMC7434570

[128] Liu F, Chen N, Gong Y, Xiao R, Wang W, Pan Z. The long non-coding RNA NEAT1 enhances epithelial-to-mesenchymal transition and chemoresistance via the miR-34a/c-Met axis in renal cell carcinoma. Oncotarget. 2017;8(38):62927–38. doi: 10.18632/oncotarget.17757 28968960 PMC5609892

[129] Wei X, Tao S, Mao H, Zhu H, Mao L, Pei W, et al. Exosomal lncRNA NEAT1 induces paclitaxel resistance in breast cancer cells and promotes cell migration by targeting miR-133b. Gene. 2023;860:147230. doi: 10.1016/j.gene.2023.147230 36717039

[130] Xu W-W, Jin J, Wu X-y, Ren Q-L, Farzaneh M. MALAT1-related signaling pathways in colorectal cancer. Cancer cell international. 2022;22(1):126. doi: 10.1186/s12935-022-02540-y 35305641 PMC8933897

[131] Zhang J, Li Q, Xue B, He R. MALAT1 inhibits the Wnt/β-catenin signaling pathway in colon cancer cells and affects cell proliferation and apoptosis. Bosnian journal of basic medical sciences. 2020;20(3):357–64. 31733641 10.17305/bjbms.2019.4408 PMC7416178

[132] Xu J, Xiao Y, Liu B, Pan S, Liu Q, Shan Y, et al. Exosomal MALAT1 sponges miR-26a/26b to promote the invasion and metastasis of colorectal cancer via FUT4 enhanced fucosylation and PI3K/Akt pathway. Journal of experimental & clinical cancer research: CR. 2020;39(1):54. doi: 10.1186/s13046-020-01562-6 32209115 PMC7092616

[133] Zhang C, Yao K, Zhang J, Wang C, Wang C, Qin C. Long Noncoding RNA MALAT1 Promotes Colorectal Cancer Progression by Acting as a ceRNA of miR-508-5p to Regulate RAB14 Expression. BioMed research international. 2020;2020:4157606. doi: 10.1155/2020/4157606 33344634 PMC7732393

[134] Wang H, Huang C, Yao X. The functions of long non-coding RNAs in colorectal cancer. Translational cancer research. 2019;8(5):2192–204. doi: 10.21037/tcr.2019.08.23 35116969 PMC8797667

[135] Wang N, Hou MS, Zhan Y, Shen XB, Xue HY. MALAT1 promotes cisplatin resistance in cervical cancer by activating the PI3K/AKT pathway. European review for medical and pharmacological sciences. 2018;22(22):7653–9. doi: 10.26355/eurrev_201811_16382 30536307

[136] Fang Z, Chen W, Yuan Z, Liu X, Jiang H. LncRNA-MALAT1 contributes to the cisplatin-resistance of lung cancer by upregulating MRP1 and MDR1 via STAT3 activation. Biomedicine & pharmacotherapy = Biomedecine & pharmacotherapie. 2018;101:536–42. 29505924 10.1016/j.biopha.2018.02.130

[137] Li P, Zhang X, Wang H, Wang L, Liu T, Du L, et al. MALAT1 Is Associated with Poor Response to Oxaliplatin-Based Chemotherapy in Colorectal Cancer Patients and Promotes Chemoresistance through EZH2. Molecular cancer therapeutics. 2017;16(4):739–51. doi: 10.1158/1535-7163.MCT-16-0591 28069878

[138] Wei Z, Zhou J, Yu H, Pu Y, Cheng Y, Zhang Y, et al. Zuo Jin Wan Reverses the Resistance of Colorectal Cancer to Oxaliplatin by Regulating the MALAT1/miR-200s/JNK Signaling Pathway. Evidence-based complementary and alternative medicine: eCAM. 2022;2022:3032407. doi: 10.1155/2022/3032407 36248422 PMC9568309

[139] Zhang P, Zhou H, Lu K, Lu Y, Wang Y, Feng T. Exosome-mediated delivery of MALAT1 induces cell proliferation in breast cancer. OncoTargets and therapy. 2018;11:291–9. doi: 10.2147/OTT.S155134 29386907 PMC5767090

[140] Wang M, Sun X, Yang Y, Jiao W. Long non‐coding RNA OIP5‐AS1 promotes proliferation of lung cancer cells and leads to poor prognosis by targeting miR‐378a‐3p. Thoracic cancer. 2018;9(8):939–49. doi: 10.1111/1759-7714.12767 29897167 PMC6068457

[141] Liu Y, Fu X, Wang X, Liu Y, Song X. Long non‑coding RNA OIP5‑AS1 facilitates the progression of ovarian cancer via the miR‑128‑3p/CCNG1 axis. Mol Med Rep. 2021;23(5):388. doi: 10.3892/mmr.2021.12027 33760168 PMC8008222

[142] Ren X, He J, Qi L, Li S, Zhang C, Duan Z, et al. Prognostic and clinicopathologic significance of long non-coding RNA opa-interacting protein 5-antisense RNA 1 in multiple human cancers. Artificial cells, nanomedicine, and biotechnology. 2020;48(1):353–61. 31899963 10.1080/21691401.2019.1709854

[143] Tao Y, Wan X, Fan Q, Wang Y, Sun H, Ma L, et al. Long non-coding RNA OIP5-AS1 promotes the growth of gastric cancer through the miR-367-3p/HMGA2 axis. Digestive and liver disease: official journal of the Italian Society of Gastroenterology and the Italian Association for the Study of the Liver. 2020;52(7):773–9. doi: 10.1016/j.dld.2019.11.017 31959478

[144] Ghafouri-Fard S, Dashti S, Farsi M, Hussen BM, Taheri M. A review on the role of oncogenic lncRNA OIP5-AS1 in human malignancies. Biomedicine & Pharmacotherapy. 2021;137:111366. doi: 10.1016/j.biopha.2021.111366 33601149

[145] Liang J, Tian XF, Yang W. Effects of long non-coding RNA Opa-interacting protein 5 antisense RNA 1 on colon cancer cell resistance to oxaliplatin and its regulation of microRNA-137. World journal of gastroenterology. 2020;26(13):1474–89. doi: 10.3748/wjg.v26.i13.1474 32308348 PMC7152514

[146] Song L, Zhou Z, Gan Y, Li P, Xu Y, Zhang Z, et al. Long noncoding RNA OIP5-AS1 causes cisplatin resistance in osteosarcoma through inducing the LPAATβ/PI3K/AKT/mTOR signaling pathway by sponging the miR-340-5p. Journal of cellular biochemistry. 2019;120(6):9656–66. 30548308 10.1002/jcb.28244

[147] Yu Q, Li Y, Peng S, Li J, Qin X. Exosomal-mediated transfer of OIP5-AS1 enhanced cell chemoresistance to trastuzumab in breast cancer via up-regulating HMGB3 by sponging miR-381-3p. Open medicine (Warsaw, Poland). 2021;16(1):512–25. doi: 10.1515/med-2021-0249 33821219 PMC8010158

[148] Xie L, Wang L, Zhu W, Zhao J, Guo X. Editorial: Bioinformatics tools (and web server) for cancer biomarker development, volume II. Frontiers in Genetics. 2022;13. doi: 10.3389/fgene.2022.959159 36299589 PMC9589408

[149] Liu X-Y, Mei X-Y. Prediction of drug sensitivity based on multi-omics data using deep learning and similarity network fusion approaches. Frontiers in Bioengineering and Biotechnology. 2023;11. doi: 10.3389/fbioe.2023.1156372 37139048 PMC10150883

