# Supplementary figures and images for: Identification of key lncRNAs associated with oxaliplatin resistance in colorectal cancer cells and isolated exosomes: From In-Silico prediction to In-Vitro validation
Source: PLoS One. 2024 Oct 14;19(10):e0311680. doi: 10.1371/journal.pone.0311680 (PMC11472961; doi:10.1371/journal.pone.0311680)

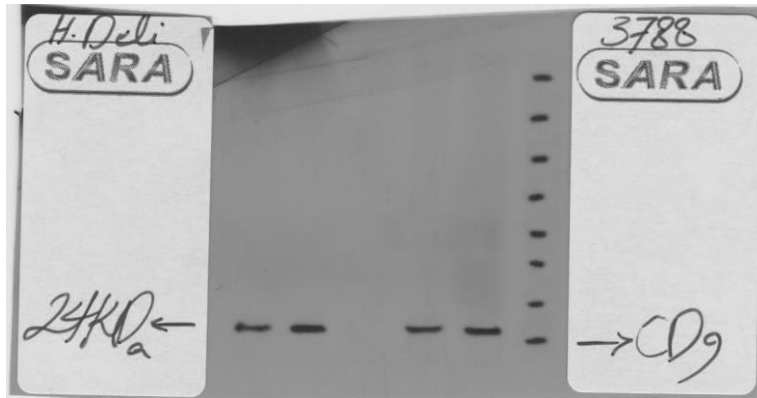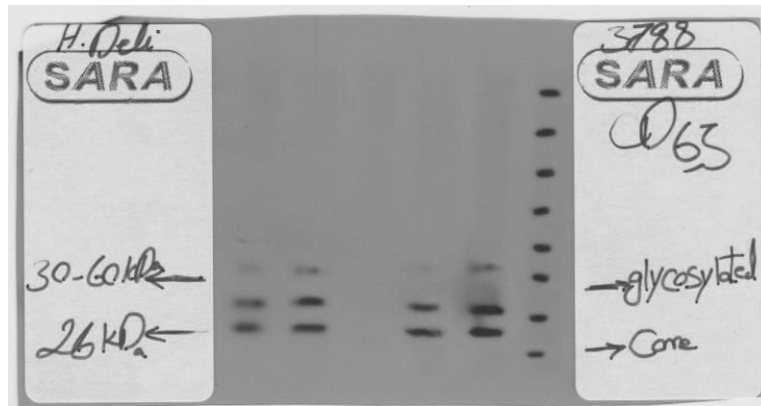

Supplement: S1 Raw images — (PDF) [file pone.0311680.s009.pdf]
