# Supplementary material for: Identification of key lncRNAs associated with oxaliplatin resistance in colorectal cancer cells and isolated exosomes: From In-Silico prediction to In-Vitro validation
Source: PLoS One. 2024 Oct 14;19(10):e0311680. doi: 10.1371/journal.pone.0311680 (PMC11472961; doi:10.1371/journal.pone.0311680)

HORIBA Scientific

HORIBA SZ-100 for Windows [Z Type] Ver2.20

SZ-100

Measurement Results

Date	:	Monday, 14 November, 2022
Measurement Type	:	Particle Size
Sample Name	:	14010823-Sahebnasagh-C-Size
Scattering Angle	:	90
Temperature of the Holder	:	25.3 °C
Dispersion Medium Viscosity	:	0.890 mPa·s
Transmission Intensity before Meas.	:	29311
Distribution Form	:	Standard
Distribution Form(Dispersity)	:	Polydisperse
Representation of Result	:	Number(Rayleigh)
Count Rate	:	15 kCPS

Calculation Results

Peak No.	S.P.Area Ratio	Mean	S. D.	Mode
1	1.00	93.8 nm	19.4 nm	81.9 nm
2	---	--- nm	--- nm	--- nm
3	---	--- nm	--- nm	--- nm
Total	1.00	93.8 nm	19.4 nm	81.9 nm

Cumulant Operations

Z-Average	:	212.1 nm
PI	:	0.796

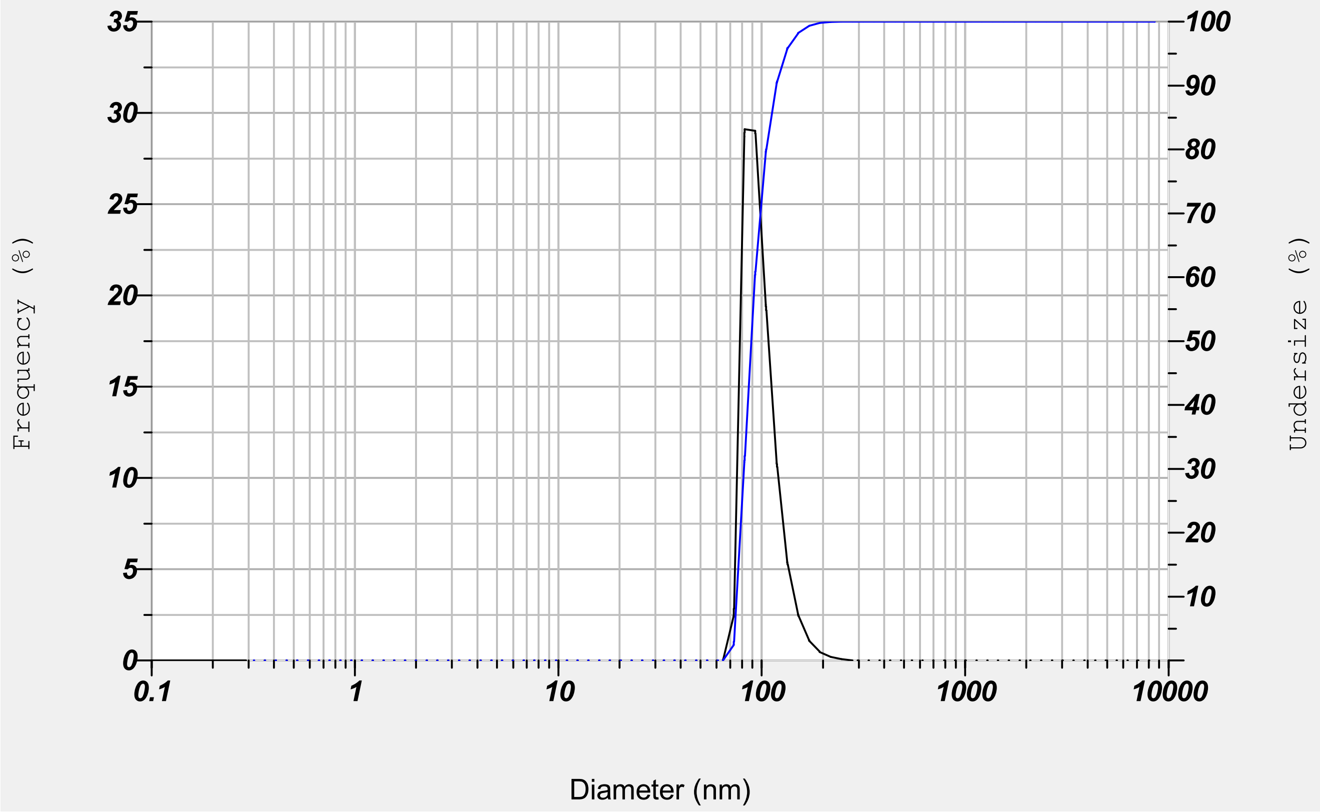

Supplement: S3 Raw images — (PDF) [file pone.0311680.s011.pdf]
